# Dietary intake, nutrition knowledge, and behavior in youth team sport athletes: a scoping review and call to advance research and practice

**DOI:** 10.1080/15502783.2026.2671431

**Published:** 2026-05-14

**Authors:** Neil Irwin, Ciarán Ó Catháin, Cormac Ward, David T. Kelly

**Affiliations:** a Department of Sport and Health Science, Technological University of the Shannon, Athlone, Ireland; b SHE Research Group, Technological University of the Shannon, Athlone, Ireland; c Department of Sport Science and Nutrition, Maynooth University, Maynooth, Ireland

**Keywords:** youth athletes, adolescent, team sports, dietary intake, nutrition knowledge, scoping review

## Abstract

**Background:**

Youth team sport athletes face unique nutritional challenges due to the combined demands of growth, development and exercise. This scoping review systematically mapped the literature on dietary intake, nutrition knowledge, education interventions and factors influencing dietary behaviors in male and female team sport athletes aged 12–19 years. The review aimed to identify knowledge gaps, highlight methodological limitations and inform recommendations for advancing both research and practice in this population.

**Methods:**

The review followed PRISMA-ScR guidelines and was pre-registered on the Open Science Framework. Eligible studies examined dietary intake, nutrition knowledge, education interventions and factors influencing dietary behavior in youth team sport athletes. Searches were conducted across PubMed, MEDLINE, PsycINFO, SPORTDiscus and gray literature sources. Study selection and data charting were conducted by the lead reviewer, with independent verification by a second reviewer. Findings were synthesized using descriptive analysis and thematic synthesis across these four domains.

**Results:**

Fifty-seven studies involving 4,369 youth team sport athletes were included. Cross-sectional designs predominated (59.6%) and the evidence base was characterized by significant methodological inconsistencies, including wide variation in dietary assessment methods, limited adjustment for misreporting and substantial underrepresentation of female athletes who accounted for 26.9% of the total sample. Energy and carbohydrate intake were frequently reported below recommended levels, most evident during periods of intensive training and competition. However, findings from studies using doubly labeled water suggest that the magnitude of reported deficits may be overestimated due to dietary underreporting and imprecise estimation of energy expenditure. Protein and fat intake generally met recommendations although micronutrient intake often fell short, particularly for vitamins A, D, and E, calcium and iron. Nutrition knowledge was limited, particularly for sport-specific topics and none of the questionnaires used were validated for adolescent populations. Twelve education interventions were identified; education-only approaches rarely produced changes in dietary behavior, whereas interventions incorporating behavior change components tended to report improvements. Only five studies examined factors influencing dietary behavior, finding that parents, coaches and peers acted as both enablers and barriers to dietary adherence, and that environmental constraints compromised dietary quality.

**Conclusions:**

Youth team sport athletes commonly exhibit inadequate reported energy and carbohydrate intake alongside frequent micronutrient shortfalls, although the reliability of these estimates is limited by methodological constraints. Nutrition knowledge is generally poor, particularly in sport-specific areas. Dietary behaviors are shaped by individual, social and environmental influences that extend beyond knowledge alone and education interventions rarely produce sustained dietary change without the integration of behavior change strategies. Future research should prioritize more rigorous and consistent methodological approaches, improved representation of female athletes and the development of youth-specific validated assessment tools. Given the scope and persistence of the methodological inconsistencies identified, the development of an expert-led consensus statement on standards for dietary assessment, nutrition knowledge measurement, intervention design and participant characterization may help advance the quality and comparability of future research in this population.

## Introduction

Adolescence is a critical period of growth and maturation, characterised by rapid physical, hormonal and neurological development [1]. During this stage, nutritional requirements increase substantially to support growth and physiological development, with additional demands placed on those engaged in regular sport participation [1,2]. By late adolescence, individuals accrue approximately 90–95% of adult bone mineral density, making adequate calcium, vitamin D and energy intake during this period essential for skeletal development and long-term musculoskeletal health [3]. For youth athletes in team sports, nutritional demands are further heightened by the frequency and intensity of training and competition, alongside the physical demands of sport-specific roles [4,5]. These demands are compounded by environmental and logistical factors common in team sport settings, including seasonal fluctuations in training load and the competing time demands of school, sport, and travel, which can limit opportunities for adequate food access and disrupt meal timing [4,5]. Despite these demands, many youth athletes lack access to professional nutrition support or structured dietary guidance [6].

Nutrition plays a vital role in fuelling training and competition and in supporting recovery processes such as glycogen resynthesis and muscle repair, all of which underpin health, adaptation and injury prevention [7,8]. In youth athletes, energy demands are amplified by the dual pressures of growth and exercise [1,2]. In elite academy soccer players, total energy expenditure ranged from 2,275 to 5,172 kcal/day across age groups, with some individuals exceeding the energy expenditure previously reported in adult professional players [2,9], highlighting the substantial energy demands placed on this population. Yet many youth team sport athletes fail to meet energy and carbohydrate recommendations, particularly during intensive training and competition phases [10–12]. These shortfalls may reflect limited nutrition knowledge, constrained fuelling opportunities and inadequate periodisation of energy and carbohydrate intake relative to training load [10,13]. Prolonged low energy availability is the recognised underlying mechanism of Relative Energy Deficiency in Sport (RED-S), a syndrome encompassing a broad range of adverse health and performance consequences, including impaired bone health, hormonal disruption, compromised immune function, impaired growth, menstrual dysfunction, increased injury risk, and chronic fatigue [14,15]. Given these risks, and the dietary inadequacies that underlie them, understanding the factors that shape dietary choices in youth athletes is essential. Nutrition knowledge is frequently identified as one such determinant, with higher knowledge levels linked to better diet quality [16].

However, nutrition knowledge alone is a weak predictor of dietary behaviour [17]. Adolescents typically demonstrate poor to moderate general nutrition knowledge, with particularly limited understanding of sport-specific topics [18]. Dietary behaviours are also shaped by broader individual and environmental influences, including cooking ability, the home food environment, time constraints and the influence of parents, peers and coaches [6,18–21]. These influences collectively demonstrate that improvements in nutrition knowledge cannot be assumed to translate into dietary behaviour change and that effective interventions in youth team sport settings must account for the broader individual and environmental factors that shape food choices.

While previous reviews have explored adolescent nutrition needs [5], dietary patterns and their determinants [22], or the relationship between nutrition knowledge and dietary intake [17], these have typically examined adult or general athlete populations and addressed these constructs in isolation. To date, no review has synthesised evidence across dietary intake, nutrition knowledge, education interventions and influencing factors specifically in youth team sport athletes. To address this gap, this scoping review systematically maps the available literature across these domains, with the aim of identifying knowledge gaps, highlighting methodological limitations and informing recommendations to advance both research and practice in this population.

## Materials and methods

### Protocol and registration

This review was conducted in accordance with the PRISMA-ScR (Preferred Reporting Items for Systematic Reviews and Meta-Analyses extension for Scoping Reviews) guidelines [23], with additional methodological guidance from the Joanna Briggs Institute framework [24] to strengthen rigour and reporting transparency. The protocol was pre-registered on the Open Science Framework (OSF) before data collection, outlining the objectives, eligibility criteria, data sources, search strategy, data charting procedures and analysis plan. The pre-registration is publicly available at https://osf.io/bd65c [25].

### Identification of the research question

The research question for this scoping review was developed using the Population, Concept, and Context (PCC) framework [24], with a specific focus on youth team sport athletes. The review aimed to address the following question: *What is the current state of research on dietary intake, nutrition knowledge and influencing factors among male and female youth team sport athletes aged 12–19?*

### Inclusion and exclusion criteria

Eligibility criteria were guided by the SPIDER (Sample, Phenomenon of Interest, Design, Evaluation, Research Type) framework [26] to accommodate the wide range of study designs and outcomes in this field. Detailed inclusion and exclusion criteria, including specifications for population, study design, topic and publication type, are summarised in Table 1.

**Table 1.** Inclusion and exclusion criteria for study selection.

Criteria	Inclusion	Exclusion
Population	Male and female team sport athletes aged 12–19 years at any competition level	Studies where data for 12–19-year-old team sport athletes could not be isolated from mixed-age or mixed-sport samples
Topic	Research addressing dietary intake, nutrition knowledge, education interventions, or factors influencing dietary behaviour in youth team sport athletes	Studies limited to isolated nutrient analysis or supplement use without examining broader dietary patterns or behaviour-related outcomes
Study design	Original research, including cross-sectional, observational, controlled trials, and randomised or non-randomised interventions	Reviews, protocols, or studies lacking sufficient methodological detail to assess study design, data collection, or analysis quality
Evaluation tools	Studies using validated, non-validated, or study-specific tools to assess nutrition knowledge or dietary intake (e.g., 24-hour recalls, food frequency questionnaires, or food diaries). Educational interventions with pre/post-assessments were included	Studies that relied solely on informal or anecdotal data, lacked defined data collection tools, or did not report any nutrition knowledge or dietary intake outcomes
Publication type	Peer-reviewed journal articles, conference proceedings, theses, dissertations, and grey literature	Sources lacking scientific review, methodological transparency, or empirical data, such as media articles, blogs, or editorial commentaries
Language and availability	Full-text studies published in English	Studies not published in English or lacking accessible full-text, limiting assessment of eligibility or methodological quality

### Search strategy and data sources

The search strategy was developed in consultation with a university librarian and peer-reviewed using the PRESS (Peer Review of Electronic Search Strategies) checklist [27]. Key benchmark studies were identified and an initial search strategy was drafted and pilot tested in PubMed to assess the sensitivity of the search and its ability to retrieve relevant benchmark studies. Age-related filters were avoided because pilot testing demonstrated that they excluded relevant studies that met the inclusion criteria. Instead, age was accounted for during study screening.

**Figure 1.**
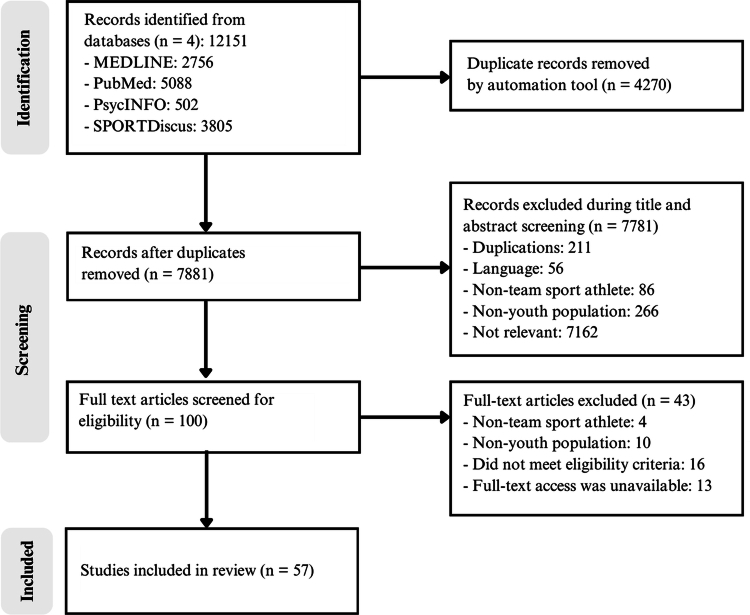
PRISMA-ScR flow diagram illustrating the study selection process.

The literature search followed the three-step process recommended by the Joanna Briggs Institute [28]: (1) an initial limited search of PubMed to identify relevant studies; (2) analysis of keywords and indexing terms to refine the strategy; and (3) application of the refined strategy across all selected databases and grey literature sources. The final strategy was applied to PubMed, SPORTDiscus, PsycINFO and MEDLINE, which were chosen to capture evidence across sport performance, nutrition, behavioural science and youth athlete development. Grey literature sources were also searched, including institutional repositories and discipline-specific archives. No restrictions were placed on publication date. All searches were conducted between January 15 and June 7, 2024. Full database strategies are provided in Appendix A. In total, the searches yielded 12,151 records, which were carried forward into the screening process (see Figure 1).

### Study selection

Study selection was conducted in accordance with PRISMA-ScR recommendations [23]. Covidence software [29] was used to identify and automatically remove duplicate records and to manage the screening process. Two reviewers (NI and CW) independently screened titles and abstracts against the predefined criteria. Full texts of potentially eligible studies were then assessed independently by both reviewers. Discrepancies were resolved through discussion, with a third reviewer (DK) consulted if consensus could not be reached. The selection process, including screening, eligibility assessment and final inclusion is detailed in the PRISMA-ScR flow diagram (Figure 1).

### Data charting

Data charting was initially informed by discussion within the research team regarding the variables to be captured in line with the review aims and objectives. The lead reviewer (NI) then extracted data from all included papers into broad charting tables. Following review of the charted data by the research team, the charting framework was refined to improve clarity, relevance and alignment with the predefined objectives of the review. In line with JBI recommendations for independent verification of data charting [28], a secondary reviewer (CW) independently charted data from a sample of included papers (*n* = 5) and this was compared with the lead reviewer's extraction to assess accuracy, consistency and alignment with the final charting framework.

### Critical appraisal of evidence

Although critical appraisal is not mandatory in scoping reviews, it was included in this review to provide additional insight into the quality of the available evidence. No studies were excluded on the basis of quality appraisal, as the purpose of the review was to map the breadth and characteristics of the available evidence. Instead, quality ratings were used to support interpretation of the findings and to contextualise the methodological strengths and limitations of the included studies. All included studies were appraised using the Academy of Nutrition and Dietetics Quality Criteria Checklist [30]. This tool assesses study quality using four relevance criteria and ten validity criteria, addressing factors such as selection bias, group comparability, clarity of intervention or exposure description, validity of outcome measures and appropriateness of statistical analysis. Two reviewers (NI and CW) independently appraised all studies and reached full agreement; consultation with a third reviewer (DK) was available but not required. Studies received a positive rating if they met at least six of ten validity criteria, with mandatory “yes” ratings for selection bias, comparability of groups, intervention/exposure description, and validity/reliability of outcome measures. Individual quality ratings for included studies are presented in Table 2.

**Table 2.** Study characteristics and participant demographics.

Study (author[s], year)	Country	Study design classification	Study quality rating (ANDQCC)	Statistical power reported	Participant characteristics (*n*, gender, age range [R], or mean age [M])	Sport, level of participation, and setting
Bell et al. [31]	Canada	Cross-Sectional Study	Positive	Not Reported	*n* 58 (Females: 43; Males: 15); R: 14 - 17	Volleyball, Trained/Developmental Level, Club
Boisseau et al. [32]	France	Cross-Sectional Study	Positive	Not Reported	*n* 11 (Males: 11); M: 15 ± 0.0	Soccer, Trained/Developmental Level, Club
Braun et al. [33]	Germany	Cross-Sectional Study	Positive	Not Reported	*n* 56 (Females: 56); M: 14.8 ± 0.7	Soccer, Highly Trained/National Level, National Team
Briggs et al. [34]	United Kingdom	Cross-Sectional Study	Positive	Not Reported	*n* 10 (Males: 10); M: 15.4 ± 0.3	Soccer, Highly Trained/National Level, Academy
Burrows et al. [35]	Australia	Cross-Sectional Study	Neutral	Not Reported	*n* 25 (Males: 25); R: 14 - 18	Rugby, Trained/Developmental Level, Regional
Caccialanza et al. [36]	Italy	Longitudinal Study	Positive	Not Reported	*n* 43 (Males: 43); R: 15 - 17	Soccer, Highly Trained/National Level, Club
Carney et al. [19]	United Kingdom	Qualitative Case Study	Neutral	Not Relevant	*n* 10 (Males: 10); R: 12 - 18	Soccer, Highly Trained/National Level, Academy
Carter et al. [6]	United Kingdom	Qualitative Case Study	Positive	Not Relevant	*n* 13 (Males: 13); M: 18 ± 1.3	Soccer, Highly Trained/National Level, Academy
Carter et al. [37]	United Kingdom	Cross-Sectional Study	Positive	Not Reported	*n* 24 (Males: 24); M: 18 ± 1.6	Soccer, Highly Trained/National Level, Club
Chapman et al. [38]	United States	Quasi-Experimental Study	Negative	Not Reported	*n* 72 (Females: 72); R: 14 - 18	Softball, Trained/Developmental Level, School
Chen et al. [39]	China	Cross-Sectional Study	Neutral	Not Reported	*n* 279 (Females: 105; Males: 174); R: 12 - 17	Soccer, Highly Trained/National Level, School
Costello et al. [10]	United Kingdom	Longitudinal Observational Study	Positive	Not Reported	*n* 6 (Males: 6); R: 16 - 18, M: 17 ± 1.0	Rugby, Elite/International Level, Club
Costello et al. [40]	United Kingdom	Quasi-Experimental Study	Positive	Not Reported	*n* 1 (Males: 1); M: 18 ± 0.0	Rugby, Elite/International Level, Club
Debnath et al. [41]	India	Cross-Sectional Study	Positive	Not Reported	*n* 90 (Males: 90); M: 16.5 ± 1.5	Hockey, Soccer, Trained/Developmental Level, Regional
Debnath et al. [42]	India	Randomised Controlled Trial	Positive	Not Reported	*n* 40 (Males: 40); M: 15.6 ± 1.8	Soccer, Highly Trained/National Level, Regional
Debnath et al. [43]	India	Quasi-Experimental Study	Neutral	Not Reported	*n* 88 (Males: 88); M: 16.2 ± 1.7	Soccer, Hockey, Highly Trained/National Level, Regional
Elliott et al. [44]	Australia	Qualitative Case Study	Negative	Not Relevant	*n* 52 (Males: 52); R: 12 - 13	Australian Football, Trained/Developmental Level, Club
Escribano-Ott et al. [45]	Spain	Cross-Sectional Study	Neutral	Not Reported	*n* 69 (Females: 37; Males: 32); R: 15 - 18	Basketball, Trained/Developmental Level, Club
Fernández-Álvarez et al. [46]	Spain	Randomised Controlled Trial	Neutral	Not Reported	*n* 319 (Males: 319); R: 13 - 16	Soccer, Trained/Developmental Level, Club
Gao et al. [47]	China	Quasi-Experimental Study	Neutral	Yes	*n* 41 (Males: 41); M: 15 ± 0.4	Soccer, Trained/Developmental Level, Club
Garrido et al. [48]	Spain	Cross-Sectional Study	Positive	Not Reported	*n* 62 (Males: 62); R: 13 - 19	Soccer, Highly Trained/National Level, Club
Ghiasvand et al. [49]	Iran	Cross-Sectional Study	Neutral	Not Reported	*n* 100 (Females: 34; Males: 66); R: 12 - 17	Basketball; Soccer; Volleyball, Highly Trained/National Level and Elite/International Level, National Team
Gibson et al. [50]	Canada	Cross-Sectional Study	Positive	Not Reported	*n* 33 (Females: 33); R: 14 - 17, M: 15.7 ± 0.7	Soccer, Highly Trained/National Level, Club
Grabia et al. [51]	Poland	Quasi-Experimental Study	Positive	Yes	*n* 46 (Males: 46); R: 14 - 16	Soccer, Highly Trained/National Level, Academy
Granja et al. [52]	Portugal	Cross-Sectional Study	Neutral	Not Reported	*n* 10 (Males: 10); M: 15.8 ± 0.4	Soccer, Highly Trained/National Level, Club
Hannon et al. [9]	United Kingdom	Cross-Sectional Study	Positive	Not Reported	*n* 24 (Males: 24); U12/13s M: 12.2 ± 0.4, U15s M: 15.0 ± 0.2, U18s M: 17.5 ± 0.4	Soccer, Highly Trained/National Level, Academy
Hickson et al. [53]	United States	Cross-Sectional Study	Neutral	Not Reported	*n* 12 (Males: 12); M: 16.4 ± 0.7	Basketball, Trained/Developmental Level, School
Iglesias-Gutiérrez et al. [54]	Spain	Cross-Sectional Study	Neutral	Not Reported	*n* 33 (Males: 33); R: 14 - 16	Soccer, Highly Trained/National Level, Club
Iglesias-Gutiérrez et al. [55]	Spain	Cross-Sectional Study	Positive	Not Reported	*n* 22 (Males: 22); R: 14 - 16	Soccer, Highly Trained/National Level, Club
Koç et al. [56]	Turkey	Cross-Sectional Study	Negative	Not Reported	*n* 377 (Males: 377); R: 13 - 19	Soccer, Highly Trained/National Level, Club
Leblanc et al. [57]	France	Longitudinal Study	Neutral	Not Reported	*n* 180 (Males: 180); R: 13 - 16	Soccer, Highly Trained/National Level, Academy
Lydon et al. [58]	Ireland	Quasi-Experimental Study	Positive	Not Reported	*n* 336 (Females: 89; Males: 247); M: 16.1 ± 2.7	GAA, Trained/Developmental Level, Club
Manore et al. [59]	United States	Cross-Sectional Study	Neutral	Not Reported	*n* 535 (Females: 297; Males: 238); R: 14 - 18	Soccer, Trained/Developmental Level, School
Martinho et al. [60]	Portugal	Cross-Sectional Study	Positive	Not Reported	*n* 25 (Males: 25); R: 14 - 16, M: 15.3 ± 0.3	Soccer, Trained/Developmental Level, Club
McHaffie et al. [11]	United Kingdom	Cohort Study	Positive	Not Reported	*n* 23 (Females: 23); M: 17.9 ± 0.5	Soccer, Elite/International Level, National Team
Nagayama et al. [61]	Japan	Longitudinal Study	Neutral	Not Reported	*n* 11 (Males: 11); M: 17 ± 1.0	Rugby, Highly Trained/National Level, National Team
Naughton et al. [62]	United Kingdom	Cross-Sectional Study	Positive	Not Reported	*n* 59 (Males: 59); U13/14s M: 12.7 ± 0.6, U15/16s M: 14.4 ± 0.5, U18s M: 16.4 ± 0.5	Soccer, Highly Trained/National Level, Academy
Naughton et al. [63]	United Kingdom	Cross-Sectional Study	Positive	Not Reported	*n* 59 (Males: 59); U13/14s M: 12.7 ± 0.6, U15/16s M: 14.4 ± 0.5, U18s M: 16.4 ± 0.5	Soccer, Highly Trained/National Level, Academy
Nikić et al. [64]	Serbia	Cross-Sectional Study	Positive	Yes	*n* 57 (Males: 57); R: 15 - 16, M: 15.6 ± 0.9	Basketball, Elite/International Level, Club
Noronha et al. [65]	Brazil	Cross-Sectional Study	Positive	Yes	*n* 73 (Males: 73); R: 14 - 19, M: 17.0 ± 1.3	Soccer, Highly Trained/National Level, Club
Patton-Lopez et al. [66]	United States	Quasi-Experimental Study	Positive	Not Reported	*n* 217 (Females: 139; Males: 78); R: 14 - 19, M: 14.9 ± 0.9	Soccer, Trained/Developmental Level, School
Pavlak et al. [67]	United States	Descriptive Observational Study	Positive	Not Relevant	*n* 138 (Females: 138); R: 14 - 18	Basketball, Trained/Developmental Level, School
Rico-Sanz et al. [68]	Puerto Rico	Descriptive Observational Study	Neutral	Not Reported	*n* 8 (Males: 8); M: 17 ± 2.0	Soccer, Elite/International Level, National Team
Russell & Pennock [69]	United Kingdom	Cross-Sectional Study	Neutral	Yes	*n* 10 (Males: 10); M: 17 ± 1.0	Soccer, Highly Trained/National Level, Club
Saidi et al. [70]	France	Cross-Sectional Study	Neutral	Not Reported	*n* 42 (Males: 42); M: 16.2 ± 0.8	Rugby, Trained/Developmental Level, Regional
Sánchez-Díaz [71]	Spain	Longitudinal Study	Positive	Not Relevant	*n* 23 (Females: 10; Males: 13); M: 13.1 ± 0.4	Basketball, Highly Trained/National Level, Academy
Sánchez-Díaz et al. [72]	Spain	Cross-Sectional Study	Neutral	Not Reported	*n* 23 (Females: 10; Males: 13); Female M: 12.7 ± 0.5; Male M: 13.5 ± 0.3	Basketball, Highly Trained/National Level, Club
Scanlon & Norton [73]	Ireland	Cross-Sectional Study	Positive	Not Reported	*n* 28 (Males: 28); R: 16 - 17	Rugby, Highly Trained/National Level, Regional
Silva et al. [74]	Portugal	Cross-Sectional Study	Positive	Not Reported	*n* 19 (Females: 7; Males: 12); R: 16 - 18; M: 17 ± 0.7	Basketball, Highly Trained/National Level, National Team
Smith et al. [75]	United Kingdom	Cross-Sectional Study	Positive	Not Reported	*n* 87 (Males: 87); R: 14 - 19	Rugby, Highly Trained/National Level, Academy
Stables et al. [12]	United Kingdom	Cross-Sectional Study	Positive	Yes	*n* 14 (Males: 14); M: 13.4 ± 0.2	Soccer, Highly Trained/National Level and Trained/Developmental Level, Academy
Stokes et al. [20]	New Zealand	Qualitative Case Study	Positive	Not Relevant	*n* 20 (Males: 20); R: 16 - 18, M: 17 ± 1.0	Rugby, Highly Trained/National Level, School
Taye et al. [76]	Ethiopia	Cross-Sectional Study	Neutral	Not Reported	*n* 26 (Gender: Not reported); Age Category: U17s	Soccer, Trained/Developmental Level, Club
Tektunalı Akman et al. [77]	Turkey	Randomised Controlled Trial	Positive	Yes	*n* 83 (Females: 83); R: 15 - 18, M: 17.2 ± 2.0	Basketball; Soccer; Volleyball, Highly Trained/National Level, Club
Thivel et al. [78]	France	Cross-Sectional Study	Positive	Not Reported	*n* 14 (Males: 14); R: 15 - 16, M: 16.1 ± 0.3	Rugby, Elite/International Level, Academy
Walsh et al. [79]	Ireland	Cross-Sectional Study	Positive	Not Reported	*n* 203 (Males: 203); R: 15 - 18	Rugby, Trained/Developmental Level, School
Zeng et al. [80]	China	Randomised Controlled Trial	Neutral	Not Reported	*n* 30 (Males: 30); M: 16.8 ± 1.8	Soccer, Highly Trained/National Level, Club

Abbreviations and Notes: ANDQCC = Academy of Nutrition and Dietetics Quality Criteria Checklist. Statistical power reporting was not applicable in studies using qualitative or descriptive designs. Age categories are labelled where exact mean age was not reported.

### Data synthesis

Data synthesis followed a structured two-step approach. Firstly, a descriptive analysis mapped study and participant characteristics, including publication year, country, study design and demographics (age, sex, sport, and competition level). Athlete competition level was classified using the Participant Classification Framework [81], which standardises athletic calibre based on training volume and performance metrics, enabling participants to be grouped into tiers from recreational to international level. Second, a thematic synthesis was conducted across four predefined domains: dietary intake, nutrition knowledge, nutrition education interventions and factors influencing dietary behaviour. Findings were organised into four summary tables, one for each domain (Table 3).

**Table 3.** Dietary intake assessments and key findings in youth team sport athletes.

Study (author[s], year)	Dietary intake assessment method(s) & duration	Handling of dietary reporting bias	Dietary intake variable(s) assessed	Reported dietary intake data	Key dietary intake findings
Bell et al. (2023)	Block 2014 food frequency and activity questionnaire [82]: 127 items; reference period: previous 6-months	Not Reported	Energy intake; diet quality	EI: 1968 ± 734 kcal/day (33.5 ± 13 kcal/kg); CHO: 241 ± 99 g/day; FAT: 81 ± 29 g/day; PRO: 77 ± 33 g/day; fibre: 19.6 ± 9 g/day	Athletes met ~60% of energy needs and exceeded protein recommendations; intakes of fibre, calcium, potassium, magnesium, folate, vitamins D and E were below guidelines; fruit, vegetable, and grain consumption was inadequate
Boisseau et al. (2002)	Food records collected via questionnaire, including all weighed portions: 7-day duration	Not Reported	Energy, macronutrients and micronutrients intake	EI: 2345 kcal/day; CHO: 51% TEI; FAT: 31% TEI; PRO: 1.68 g/kg/day	Despite a 170 kcal/day surplus, carbohydrate intake was insufficient; excessive sugar intake and deficiencies in zinc, calcium, magnesium, vitamins A, B6, D, and fibre were observed
Braun et al. (2018)	Food record: 7-day duration	Applied Goldberg cut-offs to identify misreporting based on energy requirement variability	Energy, macronutrients and micronutrients intake	EI: 2262 ± 368 kcal/day (40.5 ± 7.0 kcal/kg); CHO: 5.4 ± 1.1 g/kg/day; FAT: 1.4 ± 0.4 g/kg/day; PRO: 1.4 ± 0.3 g/kg/day	75% of athletes were in negative energy balance (mean deficit: 141 kcal/day); 84% exceeded saturated fat limits; carbohydrate and protein intakes were low; common deficiencies in vitamins D, B12, folate, A, calcium, phosphorus, and iron
Briggs et al. (2015)	Weighed food diary and 24-h recall: 7-day duration	Adjusted energy intake using correction factor for under-reporting	Energy and macronutrients intake	EI: 2246 ± 321 kcal/day; CHO: 5.6 g/kg/day; FAT: 1.2 g/kg/day; PRO: 1.5 g/kg/day	Mean energy deficit was 311 kcal/day, increasing to 544 kcal on match days and 505 kcal on high-load days; protein intake was adequate; carbohydrate intake was below recommendations
Burrows et al. (2016)	120-item semi-quantitative food frequency questionnaire (FFQ) [83]: diet quality assessed via Australian Recommended Food Score	Assessed misreporting via EEI:EEE ratio and Schofield’s BEE equation; excluded implausible data	Energy, macronutrients and micronutrients intake; diet quality	EI: 2478 kcal/day; CHO: 3.6 ± 0.0 g/kg/day; FAT: 1.2 ± 0.0 g/kg/day; PRO: 1.5 ± 0.0 g/kg/day	Carbohydrate and protein met general targets but carbohydrate remained < 5–7 g/kg/day; fat intake was within range but high in saturated fat; calcium and iron exceeded needs; fibre and fruit intake were adequate; vegetable intake was low; 8% used supplements
Caccialanza et al. (2007)	Food records: 4-day duration; collected twice, three months apart	Assessed misreporting via EEI:EEE ratio and Schofield’s BEE equation; excluded implausible data	Energy, macronutrients, fibre, cholesterol and sugar intake	EI: 2600 kcal/day (37.5 kcal/kg); CHO: 5.0 g/kg/day; FAT: 87.1 g/day; PRO: 1.5 g/kg/day	Underreporting was evident (mean deficits: 890 and 810 kcal); diets were high in cholesterol, low in fibre, and lacked adequate fruit and vegetables
Carter et al. (2023)	RFPM [84]: 7-day duration with real-time clarifications; daily 24-hour recall	Not Reported	Energy and macronutrients intake	CHO (g/kg/day) across match week: MD−3 to MD + 3 = 3.3–4.2 ± 1.1–1.9	Energy and carbohydrate intake remained consistent across the match week, but carbohydrate was insufficient on match days and adjacent time points (MD−1 to MD + 1), indicating poor nutritional periodisation
Chapman et al. (1997)	24-hour dietary recall: pre- and post-intervention	Not Reported	Energy and macronutrients intake	Control: + 110 kcal (1683 → 1793 kcal/day); Experimental: –162 kcal (2054 → 1892 kcal/day)	No significant changes in dietary intake or food choices; 76% aimed to lose weight, often prioritising weight control over fuelling; 43% reported supplement use
Costello et al. (2019)	Snap-*n*-send assessment [84]: 10-day non-consecutive period; energy intake assessed via doubly labelled water (DLW) method	Not Reported	Energy, macronutrients, fibre and alcohol intake	EI: 3998 kcal/day; CHO: 5.2 g/kg/day; FAT: 1.8 g/kg/day; PRO: 2.6 g/kg/day	Rugby players in pre-season had a mean deficit of 389 kcal/day; macronutrient intakes (445 g carbohydrate, 224 g protein, 149 g fat) met recommended levels
Costello et al. (2018)	Snap-*n*-send method [84]: 4-day duration; assessments at baseline and at 12 weeks	Not Reported	Energy, macronutrients, sugar and alcohol intake	Post-intervention EI: 16.7–24.5 MJ (≈3990–5855 kcal); CHO: 440–645 g; FAT: 142–213 g; PRO: 142–331 g	Energy intake increased by 46.7%, with body mass increasing by 6.2 kg (4.8 kg fat-free mass, 1.6 kg fat); free sugar intake reduced by 67.4%; alcohol intake eliminated; no illness symptoms reported
Debnath et al. (2019)	24-hour dietary recall: 3-day duration; using a semi-structured questionnaire	Not Reported	Energy, macronutrients and micronutrients intake	EI: 2757 ± 622 kcal/day (49.2 ± 6.3 kcal/kg); CHO: 8.3 ± 2.3 g/kg/day; FAT: 1.32 ± 0.23 g/kg/day; PRO: 1.96 ± 0.5 g/kg/day	Daily intakes of energy, macronutrients, and key micronutrients (calcium, iron, sodium, potassium) were below RDA recommendations
Debnath et al. (2023)	24-hour dietary recall: semi-structured questionnaire; 3-day duration	Not Reported	Energy, macronutrients and micronutrients intake	Pre-intervention: EI: 2750 ± 256 kcal; CHO: 378 ± 43 g; FAT: 77 ± 12 g; PRO: 108 ± 7 g. Post-intervention: EI: 3363 ± 469 kcal; CHO: 484 ± 73 g; FAT: 87 ± 14 g; PRO: 130 ± 16 g.	In the intervention group, total energy intake increased by 22.3%; carbohydrate, protein, fat, and fibre intakes rose by 27.9%, 19.9%, 13.8%, and 34.5%, respectively; increases were also observed in calcium, sodium, potassium, phosphorus, iron, sulphur, and nitrogen
Debnath et al. (2024)	24-hour dietary recall questionnaire	Not Reported	Energy, macronutrients and micronutrients intake	EI, CHO, FAT, and PRO were higher in the intervention group (3333 ± 567 kcal/day; 8.44 ± 1.2 g/kg/day; 1.50 ± 0.2 g/kg/day; 2.20 ± 0.3 g/kg/day, respectively) than in the control group (2532 ± 450 kcal/day; 5.49 ± 0.8 g/kg/day; 1.30 ± 0.3 g/kg/day; 1.50 ± 0.2 g/kg/day, respectively)	Intervention group showed increased intakes of carbohydrate (31.3%), protein (15.8%), fat (15.4%), and fibre (50.9%); micronutrient improvements noted in calcium, phosphorus, iron, and magnesium; no significant dietary changes occurred in the control group
Fernández-Álvarez et al. (2020)	KIDMED questionnaire [85]: 16 questions; administered twice over 6 months	Not Relevant	Mediterranean diet adherence (0–12); categorised as low (≤3), medium (4–7), high (≥8)	KIDMED Scores: Pre-test: Overall: 6.24; Control: 6.14; Intervention: 6.34. Post-test: Overall: 6.19; Control: 5.98; Intervention: 6.39	No significant changes in KIDMED scores were observed between intervention and control groups post-intervention
Garrido et al. (2007)	Weighed food method: 5-day duration with self-reported snacks; frequency of selected items also evaluated	Not Reported	Energy, macronutrients, micronutrients, fibre and caffeine intake; meal distribution of energy	Buffet vs. menu: EI: 2740 vs. 3148 kcal/day; CHO: 4.4 vs. 5.6 g/kg/day; PRO: 1.5 vs. 1.6 g/kg/day	Menu-based diets provided more energy and carbohydrate than buffet-style diets, though both remained below recommended levels; protein targets were met, but both diets were high in fat, especially saturated fat and cholesterol
Ghiasvand et al. (2017)	24-hour dietary recalls: conducted over a 3-day period	Not Reported	Energy, macronutrients and micronutrients intake	Volleyball: M 3725 kcal, 520 CHO, 124 PRO, 126 FAT; F 2726 kcal, 356 CHO, 102 PRO, 96 FAT. Basketball: M 3878 kcal, 591 CHO, 166 PRO, 143 FAT; F 2460 kcal, 362 CHO, 100 PRO, 83 FAT. Football: M 3223 kcal, 445 CHO, 139 PRO, 106 FAT; F 3122 kcal, 445 CHO, 126 PRO, 100 FAT.	Males consumed more energy, protein, carbohydrate, and fat than females; energy and macronutrient status was adequate; however, micronutrient intake was inadequate, particularly vitamin D and folic acid
Gibson et al. (2011)	Food diary: 4-day duration	Not Reported	Energy, macronutrients and micronutrients intake	EI: 2079 ± 460 kcal/day; CHO: 5.0 ± 1.6 g/kg/day; FAT: 1.2 g/kg/day; PRO: 1.4 g/kg/day	Mean energy intake was 2,079 ± 460 kcal/day, with an average deficit of 462 ± 549 kcal; carbohydrate, protein, and fat intakes were below guidelines; deficiencies included vitamin D (50%), ferritin (89.3%), pantothenic acid (54.5%), folate (69.7%), vitamin E (100%), and calcium (66.7%)
Grabia et al. (2022)	Questionnaire-based diet assessment: pHDI-10 and nHDI-14 indices [86]; 3-day dietary interviews	Not Relevant	Dietary patterns; macronutrients, micronutrients and supplement intake	Education group: EI 2237→2107 kcal/day; CHO 5.2→4.8 g/kg/day; PRO 1.7 g/kg/day; FAT 1.1→1.0 g/kg/day. Non-education group: EI 2339→2310 kcal/day; CHO 5.1→4.7 g/kg/day; PRO 1.5→1.6 g/kg/day; FAT 1.0 g/kg/day.	Post-education, carbohydrate, fibre, and saccharose intake improved; Unhealthy Diet Index scores decreased (15 → 13); Healthy Diet Index scores increased; self-perceived proper eating rose from 41% to 83%
Granja et al. (2017)	Food diaries and photographic records: 3-week period; reporting match day and highest training load day each week	Not Reported	Energy and macronutrients intake	EI: 2656.5 kcal/day (37.75 kcal/kg); PRO: 2.05 g/kg/day; CHO: 5.2 g/kg/day; FAT: 1.0 g/kg/day	Energy and carbohydrate intakes were below recommendations; protein intake exceeded guidelines, and fat intake was within the recommended 20–35% of total energy
Hannon et al. (2020)	RFPM [84]: 7-day duration; supplemented with at least one 24-hour recall	Not Reported	Energy and macronutrients intake	U12/13: EI 2736 ± 406 kcal/day; CHO 5.5 ± 0.9; PRO 1.6 ± 0.3; FAT 1.4 ± 0.4 g/kg/day. U15: EI 2884 ± 378 kcal/day; CHO 5.6 ± 0.8; PRO 1.5 ± 0.3; FAT 1.3 ± 0.3 g/kg/day. U18: EI 3070 ± 346 kcal/day; CHO 5.7 ± 0.8; PRO 1.4 ± 0.3; FAT 1.2 ± 0.3 g/kg/day.	U18 academy players had the highest absolute protein intake; relative protein was consistent across age groups; energy intake increased with age, with U18s reporting the highest intakes (2,542–5,172 kcal/day), exceeding values observed in some adult professionals
Hickson et al. (1990)	Weighed food record: 3-day duration	Not Reported	Energy, protein and micronutrients intake	EI: 3400 ± 702 kcal/day (45 ± 10 kcal/kg); PRO: 208 ± 52% of RDA	Basketball players had higher energy and carbohydrate intake than high school football players; most micronutrient intakes exceeded RDAs, though marginal intakes (<70% RDA) were noted for zinc, calcium, and vitamin A
Iglesias-Gutiérrez et al. (2005)	Weighed food intake and food record questionnaires: 6-day duration; follow-up clarification for ambiguous data	Not Reported	Energy, macronutrients and micronutrients intake; food group contributions to daily energy and macronutrients intake	EI: 3003 kcal/day (46.5 kcal/kg); CHO: 5.6 g/kg/day; FAT: 127 g/day; PRO: 1.9 g/kg/day	Energy intake (3,003 kcal/day) slightly exceeded expenditure (2,983 kcal/day); protein exceeded recommendations; carbohydrate (45% TEI) was low and fat (38% TEI) was elevated; deficiencies noted in folate, vitamin E, calcium, magnesium, and zinc
Iglesias-Gutiérrez et al. (2008)	Food diary record: 7-day duration; follow-up clarification for ambiguous data	Not Reported	Energy and macronutrients intake; dietary habits	EI: 2915 kcal/day (47.0 kcal/kg); CHO: 6.9 g/kg/day; PRO: 1.9 g/kg/day	Protein, lipid, and cholesterol intakes exceeded guidelines; carbohydrate was low; fruit, fish, egg, and legume intakes met recommendations, but cereals, vegetables, and nuts were underconsumed; meat, dairy, and sweets were overconsumed
Koç et al. (2016)	Ad hoc general nutrition habits questionnaire	Not Relevant	General nutrition habits	Meal frequency: 45.1% consumed 3 meals/day, 33.4% consumed 4 meals/day, 12.7% consumed 2 meals/day, 8.8% consumed 5 meals/day	Only 37.9% of athletes consumed vegetables weekly; 13.3% never did; supplement use reported by 25.2%; milk and cheese consumption reported by 40.6% and 67.1%, respectively
Leblanc et al. (2002)	Food diary: 5-day duration; annual assessments over a 3-year period	Not Reported	Energy, macronutrients and micronutrients intake; meal distribution of energy	EI: 2352 ± 454 to 3395 ± 396 kcal/day; CHO: 48.5 ± 4.3% to 56.6 ± 3.1%; FAT: 29.1 ± 2.8% to 34.1 ± 3.1% TEI; PRO: 1.79 ± 0.40 to 2.30 ± 0.50 g/kg/day	Energy intake was low overall; protein and fat exceeded recommendations; carbohydrate was inadequate; calcium intake improved over three years but remained insufficient in 5 of 9 groups; lunch provided suboptimal energy; snacks contributed more than recommended
Martinho et al. (2023)	Food diary: 5-day duration; follow-up clarification for ambiguous data via individual interviews	Not Reported	Energy and macronutrients intake; meal distribution of energy and macronutrients	EI: 1928 ± 388 kcal/day; CHO: 4.0 g/kg/day; FAT: 0.9 g/kg/day; PRO: 1.9 g/kg/day; EI lower at breakfast/snacks vs. lunch/dinner	Soccer players consumed 1,928 ± 388 kcal/day, ~1,640 kcal below expenditure (3,568 kcal/day); carbohydrate and fat intakes were suboptimal; protein met guidelines but was lowest at breakfast (12.6 g) and snacks
McHaffie et al. (2023)	RFPM [87]: 10-day duration	Self-reported EI adjusted upward by 22% to correct under-reporting	Energy and macronutrients intake	EI (adjusted for under-reporting): 2505 ± 490 kcal/day (40.9 ± 9.1 kcal/kg); CHO (Match day): 4.8 ± 1.1 g/kg/day	Low energy availability (LEA) prevalence decreased from 34% to 5% after adjusting for under-reporting; carbohydrate intake improved on match days but remained below guidelines
Nagayama et al. (2019)	RFPM [84] and weighed food record: 4-day duration	Not Reported	Energy and macronutrients intake	EI: 3540 ± 407 kcal/day; CHO: 7.9 ± 1.1 g/kg/day; FAT: 1.4 ± 0.1 g/kg/day; PRO: 1.7 ± 0.2 g/kg/day	Mean energy deficit: −213 ± 505 kcal/day; carbohydrate was inadequate for 55% of participants; protein was adequate; fat was insufficient in 9%
Naughton et al. (2016)	Weighed food diary: 7-day duration; follow-up clarification for ambiguous data	Excluded 32 participants due to incomplete dietary records	Energy, macronutrients and supplement intake; meal distribution of energy and macronutrients	EI: 2690 kcal/day; CHO: 350 g/day; FAT: 99 g/day; PRO: 106 g/day	No group differences in energy, carbohydrate, or fat intake; U18s consumed more protein than U13/14s and U15/16s, meeting protein recommendations but with uneven distribution (highest at dinner, lowest at breakfast); no group met carbohydrate recommendations (6–10 g/kg/day)
Naughton et al. (2017)	Food diary record: 7-day duration; follow-up clarification for ambiguous data	Excluded 32 participants due to incomplete dietary records	Energy, carbohydrate, micronutrients and sugar intake	U13/14: EI 1903 ± 432 kcal/day; CHO 266 ± 58 g/day. U15/16: EI 1927 ± 317 kcal/day; CHO 275 ± 62 g/day. U18: EI 1958 ± 390 kcal/day; CHO 224 ± 80 g/day	Dietary practices improved with age and progression in playing level, reflected by reduced sugar intake—likely influenced by enhanced nutrition education and structured food environments; most micronutrient intakes exceeded UK RNIs, though calcium was low in U18s and potassium was low in U15/16s and U18s
Nikić et al. (2014)	Modified self-administered food frequency questionnaire (FFQ) for young athletes [88,89]: 45 questions; reference period: previous month	Not Relevant	Food group intake (e.g., cereals, fruits, vegetables, dairy)	EI 3962 ± 1376 kcal/day (51.1 ± 16.5 kcal/kg); CHO 6.3 ± 2.1 g/kg/day; FAT 165.6 ± 64.4 g/day; PRO 1.8 ± 0.7 g/kg/day; fibre 38.3 ± 13.6 g/day	32% of athletes consumed < 1.4 g/kg protein, 51% consumed > 1.7 g/kg protein, and 56% consumed < 6 g/kg carbohydrate; deficiencies noted in vitamin A (~70%), zinc (49%), niacin, and calcium (~30%)
Noronha et al. (2020)	Semi-structured dietary record: 3-day duration	Applied Goldberg cut-offs and PAL; adjusted misreporters using residual method	Energy, macronutrients and micronutrients intake; dietary habits	CHO 3.9 ± 1.0 g/kg/day; FAT 25.1 ± 9.8% TEI; PRO 1.4 ± 0.5 g/kg/day	Diets were characterised by low intakes of carbohydrate, unsaturated fats, fibre, cereals, fruits, vegetables, and dairy, alongside high intakes of oils, sweets, meat, and eggs; deficiencies observed in calcium, folate, magnesium, and vitamins C and E
Pavlak et al. (1998)	Butterworth Hospital nutritional FFQ [90]: 7-day duration	Not Relevant	General nutrition habits	Mean nutritional score: 39.25; 18.8% of athletes had adequate diet quality (criteria not defined)	81.2% of athletes had inadequate diets, marked by high sugary food and beverage intake and poor vegetable consumption
Rico-Sanz et al. (1998)	Food diary and daily dietary recall: 12-day duration	Not Reported	Energy, macronutrients and micronutrients intake	EI 3952 kcal/day; CHO 8.3 g/kg/day; FAT 142 g/day; PRO 2.3 g/kg/day	Athletes maintained a 12-day calorie surplus, met carbohydrate recommendations, and had adequate micronutrient intakes, except for slight deficiencies in calcium and vitamin A
Russell & Pennock (2011)	Diet sheets: 7-day duration; follow-up clarification for ambiguous data via individual interviews	Not Reported	Energy and macronutrients intake	EI 2831 ± 164 kcal/day; CHO 5.9 g/kg/day; FAT 1.5 g/kg/day; PRO 1.7 g/kg/day	Energy expenditure (3,618 ± 61 kcal/day) exceeded energy intake (2,831 ± 164 kcal/day) by 788 ± 174 kcal; protein intake met guidelines; fat slightly exceeded; carbohydrate intake was inadequate; fibre intake reached 67% of RNI; most micronutrients met or exceeded recommendations
Saidi et al. (2024)	Weighed recorded food method: 7-day duration	Not Reported	Energy intake	EI 3551 ± 567 kcal/day (44.3 ± 10.5 kcal/kg)	Low energy availability was present in 47.6% of players, while only 16.6% achieved optimal energy availability
Sánchez-Díaz (2022)	Modified dietary questionnaire [91]	Not Relevant	General nutrition habits	No change in nutrition habits post-intervention; 18.8% of athletes had adequate nutrition habits	No statistically significant changes were observed in food habits or nutritional intake
Sánchez-Díaz et al. (2021)	Modified dietary questionnaire [91]: 42 questions; duration not reported	Not Relevant	Food/beverage frequency (28 items); meals, fruit, vegetables, soft drinks (14 items)	95% ate breakfast daily; 78% ate 3 meals/day; inadequate fruit intake in 23% of males, 40% of females; inadequate vegetable intake in 46% of males, 70% of females.	Fruit and vegetable consumption was low: 23% of males and 40% of females rarely or never consumed fruit; 46% of males and 70% of females reported low vegetable intake
Scanlon & Norton (2024)	Estimated food intake record: 3-day duration	Applied revised Goldberg cut-offs accounting for BMR, PAL, and intake variability to identify misreporting	Energy and macronutrients intake	EI 3456 ± 740 kcal/day; CHO 4.4 ± 1.2 g/kg/day; FAT 1.4 ± 0.5 g/kg/day; PRO 2.2 ± 0.5 g/kg/day	Mean energy deficit was 693 kcal/day; carbohydrate intake was below recommendations; protein exceeded guidelines but was unevenly distributed; fat intake generally fell within recommended ranges
Silva et al. (2013)	24-hour recall: 7-day duration	Not Reported	Energy and macronutrients intake	Male: EI: 2895 ± 479 kcal/day; CHO: 365.5 ± 64.4 g/day; FAT: 93.5 ± 20.7 g/day; PRO: 135.4 ± 23.5 g/day. Girls: EI: 1807 ± 46 kcal/day; CHO: 218.8 ± 1.8 g/day; FAT: 64.1 ± 1.2 g/day; PRO: 82.0 ± 14.3 g/day.	Self-reported energy intake underestimated total energy expenditure by approximately 40%, indicating substantial underreporting of dietary intake
Smith et al. (2016)	Semi-quantitative diet diary: 4-day duration; follow-up clarification for ambiguous data	Not Reported	Energy and macronutrients intake	U16 vs. U19: EI: 2995 vs. 3366 kcal/day; CHO: 5.01 vs. 4.93 g/kg/day; PRO: 1.9 vs. 2.3 g/kg/day; FAT: 1.4 vs. 1.3 g/kg/day	U19 soccer players reported higher energy and protein intakes than U16s, partially attributable to increased supplement use; U19s also consumed more fruit and vegetables
Stables et al. (2023)	Remote food photographic method (RFPM) [84]: 7-day duration; recall used at end of reporting period	Not Reported	Energy and macronutrients intake	Academy vs. Non-Academy Athletes: EI: 2178 ± 319 vs. 1768 ± 362 kcal/day; CHO: 5.6 vs. 4.3 g/kg/day; FAT: 1.6 vs. 1.4 g/kg/day; PRO: 1.7 vs. 1.4 g/kg/day	Academy soccer players demonstrated higher total daily energy expenditure (3,380 ± 517 kcal/day) and energy intake than non-academy players, with no significant group differences in protein or fat intake
Taye et al. (2024)	General nutrition questionnaire [92]	Not Relevant	General nutrition habits	Age-related findings: A negative correlation was observed between age and off-season eating behaviours	No significant associations were observed between sources of nutritional information and dietary practices, including pre-training and pre-competition meal timing
Tektunalı Akman et al. (2024)	Food records: 3-day duration; assessments at baseline and after 6 months	Not Reported	Energy, macronutrients and micronutrients intake	Pre- vs. Post-Intervention (Intervention vs. Control): EI (kcal/day): 1740 → 2046 vs. 2051 → 2035; CHO (g/kg/day): 3.14 → 3.82 vs. 3.57 → 3.76; PRO (g/kg/day): 1.20 → 1.42 vs. 1.29 → 1.30	The intervention increased energy intake by 307 kcal/day, as well as protein, carbohydrate, and fat intake; LEA prevalence decreased from 73.3% to 46.7% in the intervention group, with no change in the control group; both groups remained deficient in carbohydrate, iron, and calcium
Thivel et al. (2015)	Weighed food method: 3-day duration; conducted one week apart	Not Reported	Energy and macronutrients intake; meal distribution of energy	Dinner EI (kcal): Rugby Group: 969 ± 145; Exercise Group: 1185 ± 199; Control Group: 777 ± 183	Energy intake significantly increased after evening exercise, with a larger rise following high-intensity cycling than rugby; despite increased intake, subjective appetite ratings (hunger, fullness, prospective consumption) remained stable
Zeng et al. (2020)	Weighed food diary: 3-day duration; conducted pre- and post- 4-week nutrition education programme	Not Reported	Energy, macronutrients and micronutrients intake; meal distribution of energy	Micronutrient changes: No significant changes were observed in EI or macronutrient intake between groups. Post-education increases in vitamin A, vitamin C, calcium, iron, and zinc were not statistically significant.	There were no pre-intervention differences in dietary quality; post-intervention, both groups increased intake of vitamins A and C, calcium, and zinc, without significant changes in energy intake, macronutrient distribution, or overall diet quality

Abbreviations: EI = energy intake; PRO = protein; CHO = carbohydrate; FAT = fat; TEI = total energy intake; RDA = Recommended Dietary Allowance; RNI = Reference Nutrient Intake; MJ = megajoules; CG = control group; IG = intervention group; M = male; F = female; MD = match day. Units: g/kg/day = grams per kilogram bodyweight per day; kcal/kg = kilocalories per kilogram bodyweight; kcal/day = kilocalories per day. Assessment Methods: FFQ = Food Frequency Questionnaire; RFPM = Remote Food Photography Method; DLW = Doubly Labelled Water. Energy Terms: EEI = Estimated Energy Intake; EEE = Estimated Energy Expenditure; BMR = Basal Metabolic Rate; BEE = Basal Energy Expenditure; PAL = Physical Activity Level; LEA = Low Energy Availability. Diet Quality Indices: KIDMED = Mediterranean Diet Quality Index for Children and Adolescents; pHDI-10 = Pro-Healthy Diet Index; nHDI-14 = Non-Healthy Diet Index.

## Results

### Study selection

**Figure 2.**
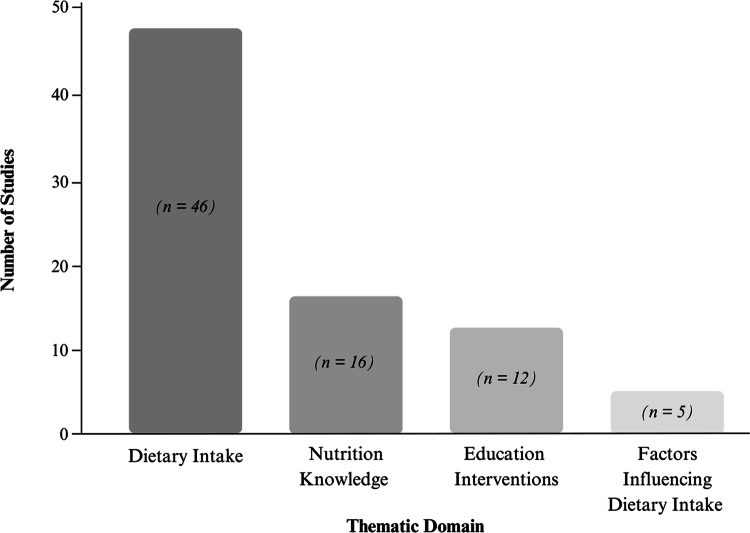
Distribution of studies across thematic domains in the scoping review.

A total of 12,151 studies were identified across four databases: MEDLINE (2,756), PubMed (5,088), PsycINFO (502) and SPORTDiscus (3,805). After removing 4,270 duplicates using Covidence software [29], 7,881 records remained and were screened by title and abstract. This stage excluded 7,781 studies based on predefined inclusion and exclusion criteria, such as additional duplicates identified manually (*n* = 211), language barriers (*n* = 56), non-team sport athletes (*n* = 86), non-youth populations (*n* = 266) and irrelevance to the review topic (*n* = 7,162). Subsequently, 100 articles underwent full-text review. Of these, 43 were excluded for the following reasons: focus on non-team sport athletes (*n* = 4), non-youth populations (*n* = 10), failure to meet other inclusion criteria (*n* = 16) and unavailability of full text despite author contact (*n* = 13). These steps resulted in 57 studies meeting the eligibility criteria and being included in the synthesis. A PRISMA-ScR flow diagram illustrates the study selection process (Figure 1) and the distribution of included studies across the four thematic domains is presented in Figure 2.

### Characteristics of studies

Most studies were published between 2020 and 2024 (42.1%), with fewer published from 2010 to 2019 (40.4%) and in 2009 or earlier (17.5%). Studies were conducted across 21 countries on six continents, with the UK (22.8%), Spain (12.3%), the USA (8.8%) and France (7.0%) contributing the most publications. Cross-sectional designs were most common (59.6%), followed by quasi-experimental studies (12.3%), randomised controlled trials (7.0%), qualitative case studies (7.0%), and longitudinal studies (8.8%). Less common designs included descriptive observational studies (3.5%) and cohort studies (1.8%). Study quality was assessed using the Academy of Nutrition and Dietetics Quality Criteria Checklist [30] and rated as positive for 34 studies, neutral for 19 and negative for 4. Only 12.3% of studies reported conducting an a priori power analysis, 77.2% did not report any power calculation and 10.5% used designs for which power analysis was not applicable.

### Characteristics of participants

A total of 4,369 youth team sport athletes were included across the 57 studies: 1,176 females, 3,167 males and 26 participants with unspecified gender. Among the studies, 70.2% focused exclusively on males, 17.5% included both genders, 10.5% focused solely on females and one study (1.8%) did not report participant gender. Participant ages ranged from 12 to 19 years. Study sample sizes ranged from 1 to 535 participants, with a median of 40 (interquartile range: 20–73).

Soccer was the most frequently studied sport (64.9%), followed by basketball (17.5%), rugby (15.8%), volleyball (3.5%) and hockey (3.5%). Less common sports included Gaelic Games, Australian football, and softball, each represented in a single study (1.8%). Athlete competition levels also varied: 54.4% of studies examined highly trained or national-level athletes, 31.6% trained or developmental-level athletes and 10.5% elite or international-level athletes. Two studies (3.5%) included participants spanning two competition levels.

### Dietary intake

Forty-six studies reported dietary intake outcomes, assessing total energy, macronutrient and micronutrient intakes and overall diet quality. Assessment duration ranged from single-day dietary recalls to multi-day food records and food frequency questionnaires that captured long-term patterns. Fourteen different dietary assessment methods were utilised, most commonly used were weighed food records (28.3%) and estimated food records (28.3%), followed by 24-hour dietary recalls (21.7%) and remote food photography (10.9%). Two or more methods were combined in 37% of studies. Reporting bias was not addressed in 60.9% of studies, deemed irrelevant in 17.4% and explicitly addressed in 21.7% (e.g., Goldberg cut-offs [6.5%], underreporting adjustments [4.3%], exclusion of incomplete records [4.3%]).

Dietary outcomes were reported in eight different formats, including absolute values (g/day), relative measures (g/kg/day), percentage of energy intake (%TEI) and adherence to recommendations (%RDA). Reported energy intake ranged from approximately 1,700 to 4,000 kcal/day, with males and older adolescents generally reporting higher intakes. Suboptimal energy intake or negative energy balance was identified in 12 of 46 studies (26.1%), most frequently during periods of high training load, though dietary underreporting may have contributed to some of these findings. Protein intake was typically adequate (1.2–2.6 g/kg/day), while fat intake (0.9–1.8 g/kg/day) generally fell within or near recommended ranges [49,93]. Carbohydrate intake varied widely (3.6–8.3 g/kg/day), with 76.9% (20/26) of studies reporting carbohydrate intake in g/kg/day showing mean values below the recommended 6–10 g/kg/day for athletes engaged in moderate to high training loads [93]. Micronutrient inadequacies were also common, most frequently involving vitamin D, calcium, iron, zinc and folate, with some studies also reporting shortfalls in vitamins A and E, magnesium and potassium.

### Nutrition knowledge

Sixteen studies assessed nutrition knowledge in youth team sport athletes, as summarised in Table 4. Four questionnaire types were used: sports nutrition knowledge (31.3%), general nutrition knowledge (31.3%), dietary and eating behaviour (25.0%) and study-specific instruments (12.5%). Questionnaire length ranged from 5 to 89 items. Half (50.0%) of the questionnaires were validated, 43.8% were not validated and 6.3% did not report validation status. None were specifically validated for adolescents. Reported knowledge scores ranged from 42% to 69.5%, with 43.8% of studies reporting poor to inadequate knowledge levels. Common knowledge gaps were identified in areas such as supplementation, nutrient timing, dietary fat and micronutrient function.

**Table 4.** Nutrition knowledge assessments and key findings in youth team sport athletes.

Study (author[s], year)	Nutrition knowledge assessment method	Nutrition knowledge assessment validation and adaptation status	Nutrition knowledge variable(s)	Nutrition knowledge data summary	Nutrition knowledge key findings
Chapman et al. (1997)	Nutrition Knowledge and Attitude Questionnaire [94]; 40 questions	Not reported	Not reported	Pre-intervention mean scores: IG = 134.8, CG = 135.7. Post-intervention: IG = 152.7, CG = 133.9	Post-education, nutrition knowledge improved; however, dietary intake remained suboptimal, particularly regarding meal composition and nutrient balance
Chen et al. (2022)	Sports Nutrition Knowledge Questionnaire (SNKQ) [95]; 66 questions	Validated (validated with adult athletes; modified)	Knowledge of food groups, micronutrients, hydration, exercise nutrition, and dietary replenishment	Total mean score: 69.5%	Higher sports nutrition knowledge was associated with more positive attitudes and healthier dietary behaviours, supporting the Knowledge-Attitude-Behaviour (KAB) model
Debnath et al. (2019)	Nutrition Knowledge Questionnaire [96]; 32 questions	Validated (validated with adolescent and young adult athletes; modified)	Protein for muscle mass; fruit and vegetable intake; fluid intake during exercise; dietary variety; meal timing; high-fat meal timing	Total mean score: 55.7%	Nutrition knowledge positively correlated with dietary practices; 62% of participants demonstrated alignment between understanding and healthier behaviours
Escribano-Ott et al. (2021)	Sports Nutrition Questionnaire [97]; 23 questions	Validated (not validated in adolescents; unaltered)	Macronutrients, hydration, recovery nutrition, weight management, and supplementation knowledge	Total mean score: 43%. Topic-specific: nutrients = 52%, hydration = 47%, recovery = 42%, weight management = 48%, supplements = 26%	Basketball players across all ages and levels demonstrated low nutrition knowledge, particularly in nutrient timing, recovery, hydration, and supplementation
Fernández-Álvarez et al. (2020)	Ad hoc self-reported survey; 5 questions	Not validated (ad hoc self-reported survey; created for this study)	Knowledge of the Mediterranean Diet and its principles	Post-intervention scores: IG = 3.42, CG = 2.53 (on 5-point scale); total mean score = 51%	Post-intervention, the intervention group showed improved knowledge of the Mediterranean diet, though pre-intervention scores were not reported
Gao et al. (2022)	Athlete Nutrition Knowledge, Attitude, and Eating Behaviour Questionnaire (KAP) [80]; 52 questions	Validated (validated in 12–25-year-olds; modified)	General and sports nutrition: energy sources, vitamins, minerals, water, fibre, meal timing, food choices, rehydration, and sports products	Pre-intervention scores: Classroom = 85%, WeChat = 84%. Post-intervention: Classroom = 88%, WeChat = 83%	Baseline nutrition knowledge was low (~61%). Both classroom-based and WeChat-delivered education improved knowledge, with greater and more sustained gains observed in the classroom group
Lydon et al. (2022)	Ad hoc pre-workshop questionnaire: 15 questions; post-workshop questionnaire: 8 questions	Not validated (ad hoc self-reported survey; created for this study)	Energy sources; macronutrient and micronutrient roles; portion sizes; fruit and vegetable servings	Total mean score: 61% (females = 71%, males = 59%)	Moderate baseline knowledge was observed; both sexes performed well on energy and fruit/vegetable intake questions, with females scoring higher overall. Post-workshop improvements were reported
Manore et al. (2017)	Sports Nutrition Knowledge Questionnaire [79]; 40 questions	Not validated (used with adolescent athletes; modified)	Hydration; pre-/post-exercise fuelling; protein and carbohydrate timing; supplement knowledge; nutrition attitudes and practices; education needs	Total mean score: 45.6% (females = 45.1%, males = 46.2%)	No sex differences were observed in knowledge, but significant disparities were found by race, ethnicity, and socioeconomic status; Latino athletes and those with lower competitive levels scored lower
Noronha et al. (2020)	Nutritional Knowledge Test [98]; 5 questions	Validated (validated with students and adolescent athletes; unaltered)	General and sports nutrition: Brazilian Food Guide Pyramid, hydration, dietary supplements, food choices, and fuelling for training	Total mean score: 54.6%. Topic-specific: BNK = 75.7%, SNK = 67.8%, FPNK = 16.3%	Moderate knowledge was observed, with 76% unaware of the Brazilian Food Pyramid; misconceptions about carbohydrate foods and limited understanding of nutrition beyond weight control were reported
Patton-Lopez et al. (2018)	Sports Nutrition Knowledge Questionnaire [79]; 40 questions	Not validated (used with adolescent athletes; unaltered)	Hydration; pre-/post-exercise nutrition; macronutrient timing; supplements; nutrition attitudes and education needs	Pre-intervention mean scores: IG = 52%, CG = 50%. Post-intervention: IG = 61%, CG = 51%	The intervention group improved sports nutrition knowledge scores by 10%, surpassing the control group; female athletes in the intervention group showed the largest gains
Sánchez-Díaz (2022)	Dietary Questionnaire on Food Habits, Eating Behaviour, and Nutrition Knowledge [91]; 11 questions	Not validated (reliability assessed in adolescents; modified)	Functions and relevance of macronutrients and micronutrients	Not reported	No statistically significant improvements in knowledge were observed following a 5-month intervention in U-14 basketball players
Sánchez-Díaz et al. (2021)	Dietary Questionnaire on Food Habits, Eating Behaviour, and Nutrition Knowledge [91]; 11 questions	Not validated (reliability assessed in adolescents; modified)	Functions and relevance of macronutrients and micronutrients	Total mean score: 42% (males = 37%, females = 47%). Topic-specific: CHO = 57%, fibre = 17%, fat = 9%, PRO = 57%, energy/calories = 48%, vitamins/minerals = 4%, balanced diet = 22%, DEE = 65%, biological foods = 57%, GM foods = 22%	Elite U-14 basketball players demonstrated poor nutrition knowledge, with no sex differences; key gaps included fibre, fat, vitamins, minerals, and balanced diet understanding
Scanlon & Norton (2024)	Nutrition for Sport Knowledge Questionnaire (NSKQ) [99]; 89 questions	Validated (not specifically validated with adolescents; unaltered)	Weight management; macronutrients and micronutrients; sports nutrition; supplementation; alcohol	Total mean score: 49.6%. Topic-specific: weight management = 53.6%, macronutrients = 55.0%, micronutrients = 44.6%, sports nutrition = 46.7%, supplements = 36.3%, alcohol = 59.4%	The cohort exhibited poor knowledge; 62.5% incorrectly identified carbohydrates as the most calorie-dense macronutrient, and only 10.7% recognised optimal protein intake; weak correlations between NSKQ scores and dietary intake were noted
Tektunalı Akman et al. (2024)	Sports Nutrition Questionnaire [97]; 83 questions	Validated (not validated in adolescents; unaltered)	Knowledge across nutrients, hydration, recovery nutrition, weight management, and supplementation	Pre-intervention mean scores: IG = 29.2, CG = 27.0. Post-intervention: IG = 35.3, CG = 27.0	The intervention improved knowledge by 7.2%, though both groups still scored below 50%, highlighting the continued need for targeted education
Walsh et al. (2011)	Sports Nutrition Knowledge Questionnaire [79]; 40 questions	Not validated (ad hoc questionnaire; created for this study)	Hydration; fuelling before/after exercise; timing of protein and carbohydrate intake; supplement knowledge; attitudes and practices	Total mean score: 59.7%. Topic-specific: hydration = 76.4%, protein = 39.2%. Awareness: 97% knew dehydration impairs performance; 39.4% recognised sports drinks are suitable for prolonged exercise	Overall nutrition knowledge was poor, with better awareness of hydration; refuelling misconceptions were common, and protein knowledge was particularly weak
Zeng et al. (2020)	Knowledge, Attitude, and Practice (KAP) Questionnaire [80]; 49 questions	Validated (validated in 12–25-year-olds; unaltered)	General and sports nutrition: energy sources, vitamins, minerals, water, fibre, meal timing, food choices, and rehydration	Pre-intervention: 45.6% answered correctly on sports nutrition knowledge; 6% scored above 75%	Pre-intervention, only 6% scored above 75% on sports nutrition knowledge; post-intervention, the intervention group improved significantly, though additional strategies are needed to translate knowledge into behaviour change and performance outcomes

Abbreviations: IG = intervention group; CG = control group; BNK = Basic Nutrition Knowledge; SNK = Sports Nutrition Knowledge; FPNK = Food Pyramid Nutrition Knowledge; CHO = carbohydrate; PRO = protein; DEE = daily energy expenditure; GM = genetically modified; KAB = Knowledge-Attitude-Behaviour; NSKQ = Nutrition for Sport Knowledge Questionnaire. Ad hoc = custom questionnaire developed for the study; not previously validated.

### Education interventions

Table 5 summarises the characteristics and outcomes of 12 education intervention studies. Delivery methods included group-based formats (91.7%), individual support, online platforms, printed materials and practical cooking or food skills sessions. Intervention duration ranged from a single 3-hour workshop to two years, with a median of 10 weeks. Most studies (83.3%) did not include follow-up beyond the immediate post-intervention period. Control groups were incorporated in 66.7% of studies, comparison groups in 16.7%, and 8.3% included neither. Five studies (41.7%) relied on education delivery alone, while seven (58.3%) incorporated one or more behaviour change components, such as goal setting, self-monitoring, practical food skills training, individualised dietary counselling or parental and coach involvement. Overall, 58.3% of studies reported improvements in nutrition knowledge, with two studies also reporting gains in cooking confidence and food preparation skills. Dietary behaviour improved in five studies (41.7%), all of which incorporated behaviour change components beyond education alone. Five studies (41.7%) found no significant changes in dietary behaviour, four of which relied on education delivery alone. One study reported no improvement in either knowledge or dietary behaviour.

**Table 5.** Characteristics and effectiveness of education interventions on dietary intake and nutrition knowledge in youth team sport athletes.

Study (author[s], year)	Intervention design and delivery methods	Intervention duration and assessment timepoints	Reported Behaviour Change Components	Sustained outcomes of intervention	Control and comparison groups	Study objective(s)	Intervention effectiveness
Chapman et al. (1997)	Group-based nutrition education delivered twice weekly (45 minutes per session) over six weeks, supported by handouts, flyers, and demonstrations on ergogenic aids, hydration, pre-competition meals, and supplementation	6 weeks; baseline, post, and follow-up	Education only	Not reported	Control group included; no separate comparison group	To evaluate the effectiveness of a sports nutrition education programme in improving nutrition knowledge	Nutrition knowledge increased by 17.9 points; no significant change in dietary intake or food choices
Costello et al. (2018b)	Combined individual and group sessions grounded in the Behaviour Change Wheel and COM-B model, including environmental restructuring, goal setting, self-monitoring, and dietary tracking, with support via WhatsApp	12 weeks; baseline and post	Goal setting; self-monitoring; feedback; parental involvement/social support; environmental restructuring; prompts/reminders; enablement	Not reported	Control group included; no separate comparison group	To increase body mass by 5 kg through improved dietary habits and correction of energy deficiency	Body mass increased by 6.2 kg (24% above target), including 4.8 kg fat-free mass, indicating high dietary quality
Debnath et al. (2023)	Twice-weekly in-person dietary counselling delivered to participants, offering tailored nutritional guidance based on established recommendations; the comparison group received no dietary input	8 weeks; baseline and post	Individualised dietary counselling; tailored dietary modification with ongoing review	Not reported	Control group included; no separate comparison group	To examine the combined effects of dietary modification and intensive training on body composition, lipid profile, and fitness	Improved dietary intake, lipid profile, and fitness in the intervention group
Debnath et al. (2024)	Eight-week individualised dietary modification programme led by a sports nutritionist, incorporating behaviour change strategies to align nutrition with training demands and personal preferences	8 weeks; baseline and post	Individualised dietary counselling; tailored dietary modification with ongoing review	Not reported	Control group included; no separate comparison group	To evaluate the effects of an eight-week dietary intervention on nutritional biomarkers	Significant increases in serum protein, haemoglobin, vitamins, and minerals (*p* < 0.001); minimal or negative changes in the control group
Fernández-Álvarez et al. (2020)	Group-based intervention incorporating behaviour change techniques and parental involvement, delivered through posters, a web app with quizzes, and gamified team-based activities	6 months; baseline and post (intervention group only)	Goal setting; action planning; self-monitoring; parental involvement/social support; information on health consequences; instructions; rewards/incentives; prompts/cues; environmental restructuring	Not reported	Control group included; no separate comparison group	To evaluate the feasibility and effectiveness of a behaviour change and education-based intervention to improve healthy eating adherence and dietary knowledge	Improved nutrition knowledge; Mediterranean Diet scores increased, but not significantly compared to the control group
Gao et al. (2022)	Twelve-week programme comprising weekly 30-minute classroom sessions (lectures and discussions) and supplementary WeChat delivery with articles, comic-style graphics, and quizzes	12 weeks; baseline, post, 6-week, and 12-week follow-up	Education only	Not reported	No control group; comparison group received WeChat-based delivery	To compare classroom-based and WeChat-based nutrition education on nutrition knowledge and self-efficacy	Nutrition knowledge and self-efficacy improved in both groups (*p* < 0.05); classroom group showed greater gains in knowledge (+20%) and KAP scores
Grabia et al. (2022)	Intervention combining personalised dietary advice and a seven-part group education series covering motivation, fuelling strategies, hydration, supplementation, and dietary adjustments	17 weeks; baseline, week 11, and week 17	Individualised dietary recommendations; follow-up review; practical instruction on energy and macronutrient planning	Not reported	No control group; comparison group received individualised support only	To evaluate the effects of personalised nutrition advice and group education on dietary habits and biochemical parameters	Reduced unhealthy food intake (*p* < 0.01); self-reported improvements in regeneration, well-being, and mental focus
Lydon et al. (2022)	Single 3-hour interactive workshop incorporating nutrition education, practical cooking skills, and behaviour change strategies	Single 3-hour session; pre and post	Practical food skills training	Not reported	No control or comparison group	To enhance nutrition knowledge, cooking confidence, and healthy eating behaviours	Improved nutrition knowledge, cooking confidence, and food preparation skills
Patton-Lopez et al. (2018)	Two-year, multi-component programme including face-to-face nutrition education, life-skills workshops, team-building sessions, and cooking demonstrations delivered by a registered dietitian	2 years; baseline, end of year 1, end of year 2	Meal/snack planning; cooking demonstrations/food tastings; role play; coach involvement; shopping on a budget/food preparation skills	Sustained improvement in sports nutrition knowledge (+10%) and dietary awareness, particularly among females	Control group included; no separate comparison group	To improve sports nutrition knowledge, attitudes, and behaviours	Sports nutrition knowledge improved by ~10%, with reported shifts toward performance-focused eating and reduced sugar intake; fuelling practices unchanged
Sánchez-Díaz. (2022)	Five monthly sessions combining in-person and online formats, led by a dietitian and focused on theoretical and practical aspects of sports nutrition and healthy eating	5 months; baseline and post	Education only	Not reported	Control group included; no separate comparison group	To investigate the effects of combined nutrition education and FIFA 11 +training on fitness, physical activity, eating habits, and nutrition knowledge	No change in eating habits or nutrition knowledge
Tektunalı Akman et al. (2024)	Six in-person lectures on energy metabolism, fuelling strategies, energy availability, and general nutrition, supported by a printed workbook for guided note-taking	6 weeks; baseline and 6-month follow-up	Education only	Initial improvements in energy intake and nutrition knowledge were not sustained at 6-month follow-up	Control group included; no separate comparison group	To increase energy availability, improve body composition, and enhance nutrition knowledge	Increased energy availability, protein intake, fat-free mass (*p* < 0.05), and nutrition knowledge
Zeng et al. (2020)	Four-week intervention using educational comic books and weekly 30-minute in-person sessions; the intervention group attended both, while the control group received comic books only	4 weeks; baseline and post	Education only	Not reported	No control group; comparison group received comic book only	To evaluate the impact of a four-week nutrition education programme on dietary knowledge, attitudes, and behaviours	General and sports nutrition knowledge improved (*p* < 0.01); no change in attitudes, behaviours, or diet quality

#### Factors influencing dietary intake

Table 6 summarises the methods and findings of five studies that investigated determinants of dietary behaviour in youth team sport athletes. Data collection methods included semi-structured interviews (60%), focus groups (20%) and self-report questionnaires (20%). Reported determinants spanned individual, social and environmental factors. At the individual level, performance goals, motivation, nutrition knowledge, cooking skills and body image concerns were identified as influences on dietary behaviour. At the social level, parental involvement emerged as both a facilitator and barrier to dietary practices, with coach and peer influence also reported. At the environmental level, limited access to professional nutrition support, time constraints, the cost and convenience of unhealthy food options, living arrangements and restricted food availability at training and competition venues were identified as barriers. Four of the five studies (80.0%) emphasised the complex interplay of these contextual and interpersonal factors in shaping dietary behaviours.

**Table 6.** Factors influencing dietary intake in youth team sport athletes.

Study (author[s], year)	Assessment method(s)	Focus of investigation	Key findings
Carney et al. (2024)	Individual semi-structured interviews (face-to-face)	Player perspectives on the role of nutrition in performance and development	Limited awareness of nutrition’s role in growth, maturation, and injury prevention, particularly under-fuelling. Parental influence and academy schedules constrained adequate dietary intake, highlighting a need for structured support
Carter et al. (2022)	Individual semi-structured interviews (online), guided by the COM-B model and Theoretical Domains Framework (TDF)	Enablers and barriers to adherence to nutritional recommendations	Nutrition knowledge and cooking skills supported healthy practices but were constrained by low motivation. High-quality food provision and access to nutrition support promoted adherence to nutritional recommendations; poor provision and limited expertise hindered it. Living arrangements influenced autonomy—supportive households aided adherence but reduced independence. Awareness of nutrition’s impact on performance and body composition, and exposure to elite role models, were key enablers
Elliott et al. (2016)	Focus groups (20–30 minutes, face-to-face)	Parental influence and the availability of unhealthy foods	Parents and coaches encouraged healthy, carbohydrate-rich pre-game meals. However, the use of unhealthy post-game rewards reinforced poor dietary habits, compounded by limited healthy options at sporting venues
Escribano-Ott et al. (2021)	Modified sports nutrition questionnaire (36 items) [97]	Dietary habits and factors influencing adherence, non-adherence, and eating behaviours	Inadequate nutrition knowledge, limited professional support, and time constraints were key barriers to adherence to dietary recommendations
Stokes et al. (2018)	Individual semi-structured interviews (face-to-face)	Perceptions and determinants of dietary habits related to health and performance	Dietary intake was influenced by performance goals, motivation, and a supportive team environment. Social media, body image, wellbeing, and peer or family habits acted as facilitators or barriers to food choices. The cost and convenience of unhealthy foods often hindered healthy eating, while awareness of nutrition’s impact on performance encouraged healthier choices, particularly on game days. Motivation to eat well declined during off-season and injury periods

## Discussion

This scoping review systematically mapped and synthesised evidence on dietary intake, nutrition knowledge, education interventions and factors influencing dietary behaviours in youth team sport athletes aged 12 to 19 years. The review aimed to identify knowledge gaps, highlight methodological limitations and inform recommendations to advance both research and practice in this population. The findings reveal substantial inconsistencies in methodological quality, dietary assessment approaches and population representation across the evidence base. The key themes and implications of these findings are discussed below.

### Methodological quality and participant representation

Research on dietary intake, nutrition knowledge and influencing factors in youth team sport athletes is characterised by significant methodological limitations and limited population diversity, most notably a substantial underrepresentation of female athletes. Research output has increased notably, with 42% of studies published between 2020 and 2024, reflecting growing interest in the field. Cross-sectional designs were most common (59.6%), offering descriptive snapshots of dietary behaviours but limiting the ability to assess change over time or establish causality. Such reliance on observational data is particularly limiting in youth populations, where the complexities of growth, maturation and metabolic adaptation during adolescence [1] underscore the need for longitudinal or repeated-measure designs to capture how these developmental processes influence energy requirements, dietary intake and nutrition knowledge. Social, psychological and environmental influences on dietary behaviour are also likely to evolve across adolescence [1] and longitudinal research is needed to understand how these shifting influences shape dietary intake at different stages of development.

Only a minority of studies employed quasi-experimental (12.3%) or randomised controlled trial (RCT) designs (7.0%), underscoring the largely descriptive nature of the current evidence base. Consequently, findings should be interpreted with caution, particularly regarding causality and long-term implications for health and performance. Methodological quality was assessed using the Academy of Nutrition and Dietetics Quality Criteria Checklist [30], which provides a structured framework for evaluating rigour and relevance. However, this tool may overestimate study quality by allowing overall positive ratings despite clear limitations, such as unvalidated dietary assessment methods, lack of bias control and reliance on self-reported data without verification. In addition, 77.2% of studies did not report a priori statistical power calculations limiting the interpretability of findings and reducing their potential generalisability. Although power analysis is not applicable to all research designs, including qualitative and exploratory approaches, its omission in studies intended to detect statistical effects may undermine confidence in null findings and reduce the overall robustness of results.

In addition to methodological concerns, limited participant diversity emerged as a key issue. A substantial gender imbalance was evident, with 70.2% of studies focusing exclusively on male athletes, 10.5% on females and only 17.5% including both. Females accounted for just 26.9% of the total sample (*N* = 4,369), emphasising the need for more inclusive research across sexes. The concentration of research within soccer (64.9%), basketball (17.5%), and rugby (15.8%) populations may also limit the generalisability of findings to athletes in other team sports [100]. More than half of studies (54.4%) focused on highly trained or national-level athletes, with limited representation of recreational or developmental-level participants. The Participant Classification Framework [81] provided a consistent approach to categorising athlete participation; however, as it was developed for adult athletes, its application to youth populations may limit classification accuracy. To improve comparability across studies, a youth-specific framework for categorising athlete calibre is warranted.

### Dietary intake assessment and reporting bias

This review identified substantial variation in dietary assessment methods across studies involving youth team sport athletes. Among the 46 included studies, 14 distinct tools were used, with dietary intake reported in eight different formats. Such variability, combined with the widespread reliance on self-reported data, limits cross-study comparability and constrains the ability to draw definitive conclusions. These methodological inconsistencies are well documented in nutrition research, where tool selection is often influenced by study design, participant characteristics and logistical considerations [101,102].

A key limitation across reviewed studies was the failure to address dietary reporting bias. Of the dietary studies reviewed, 60.9% did not adjust for under- or overreporting, a critical concern in adolescents, whose self-reported dietary data are particularly vulnerable to recall error, social desirability bias and underestimation of intake [36]. Only a minority of studies implemented strategies to address dietary misreporting, with 6.5% applying Goldberg cut-offs to identify implausible energy intake values, 4.3% applying underreporting adjustments and 4.3% excluding incomplete records. These strategies were primarily used to screen and exclude data rather than adjust dietary intake values. One study reported both unadjusted and corrected values [11], but this was an exception. The absence of standardised protocols raises concerns about the validity of reported dietary intake, undermines confidence in energy availability estimates and limits comparability across studies. Misreporting is well documented in nutrition research [35] and, when uncorrected, may lead to significant underestimation of energy and nutrient intake, particularly in youth athletes with elevated energy requirements due to growth and exercise demands [2].

Although methods for estimating energy expenditure were not examined in this review, their inherent variability, arising from fluctuating training loads, individual metabolic differences, and limitations in predictive models, presents a broader challenge for accurately assessing energy availability in youth athletes [103,104]. These methodological constraints must be considered when interpreting energy balance and low energy availability (LEA), as they may distort prevalence estimates and the perceived severity of fuelling inadequacies.

In light of these dietary assessment challenges, app-based dietary monitoring tools, including AI-assisted platforms, may offer practical advantages for youth populations by enabling real-time, image-based dietary logging that reduces recall bias and improves portion size estimation [105]. Given the feasibility and acceptability of mobile-based dietary assessment methods reported in adolescent populations [105], these approaches warrant further investigation. However, their validity, accuracy and potential influence on eating behaviours in youth athletes have not yet been established and require evaluation before widespread adoption can be recommended.

### Energy intake patterns and availability

Reported energy intake varied widely across studies, with notable differences between sexes. Across 23 studies involving 735 males, mean reported energy intake was 2,822 kcal/day (range: 1,928–3,998 kcal/day), compared with a mean of 2,231 kcal/day (range: 1,807–2,262 kcal/day) across four studies involving 119 females. These values reflect studies reporting energy intake in kcal/day, with pre-intervention values used where applicable; they do not account for age-related variation, and differences in the age distribution of male and female cohorts may have confounded comparisons between sexes. The consistently higher energy intake observed in males likely reflects greater physiological energy requirements, driven by higher fat-free mass and physical activity levels [106]. While adolescent females face similarly elevated energy demands due to growth, maturation and sport participation, the limited number of female-specific studies in this review (*n* = 4) means that current understanding of energy intake in this population remains insufficient to draw reliable conclusions. This gap reflects the broader underrepresentation of female youth athletes in the literature and highlights the need for more inclusive research to inform sex-specific nutritional strategies.

A recurring concern across studies reporting dietary intake was the prevalence of energy deficits in youth athletes, with reported shortfalls ranging from 141 to 1,640 kcal/day. The largest deficits occurred on match days and during intensive training periods, when energy expenditure increased but dietary intake did not adjust accordingly [34,60,69]. Hannon et al. [9] reported that some adolescent academy soccer players aged 13 to 18 years expended energy at levels comparable to, or exceeding, those of adult professional players, highlighting the substantial energy demands placed on youth athletes at different stages of development. Braun et al. [33] reported that 75% of athletes assessed were not meeting their estimated energy requirements, while another study found that nearly half of adolescent rugby players met the criteria for LEA using a validated threshold of < 30 kcal·kg⁻¹ FFM·day⁻¹ [70]. Collectively, these findings point to a persistent mismatch between reported energy intake and estimated expenditure in youth team sport athletes, most evident during competition and intensive training [10].

Notably, the four studies in this review that used doubly labelled water to assess energy expenditure [9,10,12,74] present a more nuanced picture than the broader evidence base suggests. Among these, one found no significant energy deficit at group level despite high energy demands [9], another identified approximately 40% underreporting of energy intake relative to measured expenditure [74] and a third reported a deficit of 389 kcal/day [10], lower than many of the deficits reported in studies relying on estimated expenditure. These findings suggest that the magnitude and prevalence of energy deficits reported across the wider literature may be overestimated due to the combined effects of dietary underreporting and imprecise estimation of energy expenditure. This concern aligns with recent critical analysis of the broader evidence base on energy availability in athletes, which has questioned the reliability of field-based energy availability assessments and highlighted the potential for overestimation of low energy availability prevalence [103]. While energy shortfalls during periods of high demand are plausible and may carry meaningful health implications, the extent to which reported deficits reflect genuine energy inadequacy, as opposed to methodological artefact, remains uncertain. Prolonged energy deficits, where they do occur, can impair growth and maturation, reduce bone mineral density, delay recovery, compromise immune function and increase injury risk [14,15,107–109].

### Macronutrient intake

Carbohydrate intake among youth team sport athletes ranged from 3.6 to 8.3 g/kg/day across studies and was frequently below recommended levels, particularly on match days and during intensive training [11,34,62,69]. Current guidelines recommend 6 to 10 g/kg/day to support high training demands [93]. However, these guidelines were developed for adult athletes and may not be directly applicable to youth populations. Youth athletes have lower glycogen storage capacity [13,110], rely more heavily on fat oxidation during submaximal exercise and may experience temporary insulin resistance during puberty due to hormonal changes that reduce glucose uptake and impair glycogen replenishment [2,111]. These developmental factors may heighten the importance of carbohydrate availability for supporting growth, recovery and training adaptation [1,2]. In adult populations, carbohydrate periodisation has been shown to enhance training adaptations and optimise performance by aligning glycogen availability with exercise demands [4,112,113], yet to the authors' knowledge no equivalent guidance exists for youth populations. This underscores the need for evidence-based, youth-specific carbohydrate guidelines that account for the metabolic differences between adolescent and adult athletes. In practice, however, youth team sport athletes often failed to align carbohydrate intake with training demands, likely contributing to energy deficits on match days and during intensive training periods [34,60,69]. Inadequate carbohydrate periodisation persists despite well-established evidence linking glycogen depletion with fatigue during intermittent exercise [13,34,114]. Insufficient fuelling on high-intensity days may impair performance, delay recovery and limit glycogen replenishment [2], while also increasing the likelihood of low energy availability [13,115]. These findings highlight the importance of practitioner-led education to help youth athletes adjust carbohydrate intake in line with day-to-day training requirements.

Protein intake among youth team sport athletes ranged from 1.2 to 2.6 g/kg/day across studies, typically meeting or exceeding adult-based recommendations of 1.5 to 2.0 g/kg/day [2,64,74]. However, these values should be interpreted in the context of overall dietary adequacy. Notably, several studies reported protein intakes consistent with or above current adult sports nutrition recommendations alongside evidence of suboptimal energy intake [9,10,36,52,57,60,61,69,73]. This suggests that adequate protein intake did not necessarily correspond with adequate overall energy intake, adding further support to the previously presented evidence of suboptimal carbohydrate intake and misalignment between carbohydrate intake and energy expenditure.

When overall energy intake is insufficient, adequate protein intake alone may not be enough to support key physiological processes such as muscle maintenance, immune function and training adaptation [14,108]. One possible explanation for this imbalance is an overemphasis on protein consumption at the expense of adequate carbohydrate intake. A recent scoping review identified similar imbalances in adult athletes, where protein intake was generally adequate or exceeded recommendations, yet carbohydrate intake was frequently suboptimal [116]. These findings reinforce the importance of evaluating protein intake within the context of overall macronutrient balance and energy availability, rather than in isolation.

Reported fat intake among youth team sport athletes generally aligned with the recommended range of 20 to 35 percent of total energy intake [4,52], with relative intake ranging from 0.9 to 1.8 g/kg/day. However, percentage-based recommendations can be misleading, particularly for athletes experiencing low energy availability, where fat may represent an appropriate proportion of total intake yet be insufficient in absolute terms to meet physiological requirements. Reporting fat intake relative to body mass (g/kg/day) may provide a more accurate basis for assessing fat intake adequacy in such cases.

Comparisons of fat intake across studies were limited by inconsistent reporting formats and methodological variation. Nevertheless, several studies raised concerns about excessive saturated fat intake. For example, Braun et al. [33] found that 84 percent of highly trained adolescent female soccer players exceeded recommended saturated fat thresholds. Similar patterns of excessive saturated fat intake, driven by frequent consumption of processed meats, confectionery and fast foods, have been reported in other adolescent soccer populations [48,55]. Although dietary fat is essential for supporting energy availability and the absorption of fat-soluble vitamins, excessive saturated fat intake may adversely affect cardiovascular and metabolic health, with potential implications for recovery and long-term performance [2,13]. This is particularly relevant during adolescence, when the body is still undergoing significant development and dietary habits are being established that may persist into adulthood [1]. Few studies have assessed fat quality or reported the relative contributions of saturated, monounsaturated, polyunsaturated and trans fats in youth team sport athletes, limiting insight into the types and sources of dietary fat consumed.

### Micronutrient intake

Seventeen of the 46 dietary studies (37%) assessed micronutrient intake. Several studies reported inadequate intake of key micronutrients, including vitamins A, D, and E, folate, calcium, iron, zinc, magnesium and potassium [31,53,63]. These findings reflect dietary insufficiencies rather than confirmed physiological deficiencies, as few studies included biomarker data. Low fruit and vegetable consumption was also common, with two studies reporting that fewer than 40 percent of athletes consumed vegetables weekly [56,72]. While not equivalent to micronutrient tracking, limited intake of plant-based foods likely contributed to observed shortfalls in micronutrient and fibre intake, particularly given the role of fruits, vegetables, legumes and whole grains in providing these nutrients [36,64].

Inadequate calcium and vitamin D intake may compromise bone mineral accrual, increasing the risk of low bone density and stress fractures during a critical growth and development period [117,118]. Iron and folate deficiencies can impair red blood cell production and oxygen transport, potentially leading to anaemia, fatigue and reduced endurance capacity [119–121]. Additionally, suboptimal intake of zinc, vitamin A, and vitamin C may weaken immune function and delay recovery [122–124]. Collectively, these findings highlight the need for targeted nutrition education and dietary strategies that promote micronutrient-dense food choices to support physiological development, reduce injury risk, and optimise health and performance outcomes in youth team sport athletes.

### Nutrition knowledge

Considerable variability was observed in the instruments used to assess nutrition knowledge across the included studies, ranging from brief, non-standardised surveys to more comprehensive, validated questionnaires. This lack of consistency hinders cross-study comparisons. While some instruments assessed general nutrition knowledge, others included sport-specific content, though none were validated for adolescent populations. This represents an important methodological limitation, as the use of instruments not validated for adolescent populations reduces confidence in the reliability, interpretability, and comparability of reported scores. Questionnaires developed for adults or general populations may not be well suited to the developmental stage, comprehension level and sport-specific context of youth athletes and may underestimate true knowledge or fail to capture the knowledge domains most relevant to this population. The recent development of youth-specific tools, such as the GESNK questionnaire [125], represents an important advancement toward improving measurement accuracy within adolescent populations.

Overall, youth team sport athletes demonstrated limited nutrition knowledge, with many studies reporting mean scores below 50% [71,73,77]. General nutrition knowledge was typically higher than sport-specific knowledge, reflecting findings reported previously [18]. Several studies reported that female athletes demonstrated higher levels of nutrition knowledge than their male counterparts [58,66,72]. Across the literature, youth athletes typically displayed a stronger understanding of energy balance, macronutrient functions and hydration. However, knowledge of dietary fats, fibre, micronutrients, supplementation and nutrient timing was consistently limited [45,58,72,73].

While foundational concepts appear moderately well understood, sport-specific topics remain poorly understood among youth athletes. Such knowledge gaps could carry implications beyond performance, with potential adverse effects on health and development, particularly given the elevated physiological demands placed on youth athletes relative to their non-athletic peers [1]. An inadequate understanding of fuelling strategies, recovery processes and micronutrient requirements may contribute to negative energy balance, impaired growth, and increased susceptibility to illness and injury [126]. The persistence of these knowledge gaps, despite repeated calls in the literature for professional support and education [36,56,59,65,73], may reflect limited resources, reliance on short-term education sessions, insufficient integration of behaviour change strategies and inadequate attention to the social and environmental contexts in which youth athletes make dietary choices. Addressing these challenges is likely to require sustained investment in practitioner-led education and coordinated action across youth sport systems.

### Education interventions

The reviewed education interventions varied considerably in design, delivery format and learning setting, spanning individual, group-based, in-person, online and hybrid approaches. Additional components used to support content delivery included quizzes, comic books and digital platforms. Intervention duration ranged from single 3-hour workshops to multi-year programmes, although most interventions lasted between 4 and 17 weeks. Shorter interventions often produced immediate improvements in nutrition knowledge [47,66,77]. However, these improvements were not consistently accompanied by changes in dietary intake [38,46,80].

Findings from the reviewed studies indicate that interventions incorporating behaviour change components beyond information delivery, such as practical food skills training, goal setting, and parental or coach involvement, tended to report improvements in dietary behaviour [40,42,43,46,51,58,66]. In contrast, education-only approaches tended to be associated with improvements in knowledge alone, without corresponding changes in dietary behaviour [38,71,80]. This emerging pattern should be interpreted with caution, given the limited number of intervention studies and the marked variation between them in duration, setting, delivery format and outcomes assessed.

Few studies examined whether intervention effects were sustained beyond the immediate post-intervention period. Among those that did, findings were mixed: one study observed that improvements in knowledge and dietary intake were no longer evident at six months following the intervention [77], whereas another reported sustained improvements in knowledge and dietary awareness over two years [66]. The absence of longer-term follow-up in most studies limits understanding of whether observed effects represent lasting behaviour change or temporary responses to the intervention period.

Taken together, the current evidence suggests that improving nutrition knowledge alone is unlikely to produce sustained dietary change in youth team sport populations. However, the evidence base does not yet provide clear guidance on which combinations of intervention content, behaviour change strategies and delivery approaches are most effective. Without this clarity, nutrition practitioners, coaches and clubs working with youth team sport populations are unable to make evidence-informed decisions about how best to invest their time and resources in supporting dietary behaviour change. Identifying which intervention features are most likely to produce lasting improvements in both nutrition knowledge and dietary practices should be a priority for future research.

### Factors influencing dietary behaviour

This review identified several interrelated factors influencing dietary behaviour in youth team sport athletes. However, only five studies specifically examined these influences, most of which were small-scale and qualitative in design. While these studies provided valuable contextual insights, the limited evidence base reinforces the need for more robust, large-scale research in this area.

At the individual level, limited nutrition knowledge and lack of professional support were linked to poor adherence to nutritional recommendations [6,45]. Cooking skills were identified as a practical enabler of healthier dietary practices [6]. However, motivation emerged as an important psychological determinant, with low motivation constraining dietary adherence even in athletes with adequate nutrition knowledge [6]. Motivation also varied by context, with athletes more likely to adhere to dietary guidelines when driven by performance goals during the competitive season, whereas adherence declined during the off-season and injury periods [6,20]. At the social level, parents, coaches and peers emerged as both enablers and barriers to healthy dietary practices [6,19,20,44]. While these groups often encouraged positive dietary behaviours, some studies reported inconsistent role modelling [44]. Supportive home environments, characterised by the availability of healthy foods and encouragement of positive eating practices, promoted greater autonomy in food preparation and healthier dietary choices [19].

At the environmental level, limited availability of healthy food options at home, during travel and in school or sport settings restricted dietary quality [19]. Time constraints and the cost and convenience of unhealthy food options further compounded these barriers [19,20,45]. Together, these findings illustrate that healthy eating in youth athletes requires not only nutritional knowledge but also the motivation, opportunity and practical capability to act upon it, further supporting the case for approaches that extend beyond athlete-directed education alone and address the broader social and environmental contexts in which youth athletes make dietary choices.

### Future research directions and best-practice considerations

Cross-sectional designs have provided valuable descriptive insights across the evidence base but cannot capture how dietary intake, nutrition knowledge and behavioural influences evolve across adolescence. Future research would benefit from greater use of longitudinal, repeated-measures and intervention designs to complement existing evidence by examining developmental change and evaluating the sustained effects of nutrition strategies. Given that growth, maturation, training demands and psychosocial influences vary across adolescence [1], future studies should account more explicitly for developmental stage when designing protocols, characterising participants and interpreting findings. The marked underrepresentation of female athletes indicates that future research should prioritise more inclusive and where appropriate, sex-stratified analyses. Greater transparency in reporting is also needed, including a priori sample size justification and detailed characterisation of participant age, sport and competition level.

Improving the rigour and consistency of dietary intake assessment is another important priority. The wide variation in assessment methods and reporting formats identified in this review limits cross-study comparability and constrains the strength of conclusions that can be drawn. The most appropriate dietary assessment method will depend on the research question, study design, population characteristics and available resources, including time, participant burden and budget. Regardless of the method selected, future studies should clearly justify their choice and report its known limitations for the population studied. Studies should also report whether implausible dietary intake values were assessed and how misreporting was handled, as unrecognised under or overreporting may compromise the validity of dietary intake findings. This is particularly critical when interpreting energy availability in youth athletes, where reliance on self-reported intake and estimated expenditure may distort prevalence estimates, as demonstrated by the doubly labelled water studies discussed earlier in this review [11,74]. Emerging technology-assisted dietary assessment tools may offer practical advantages in youth populations, but their validity, accuracy and potential influence on eating behaviours require further evaluation before widespread adoption can be recommended. Similarly, nutrition knowledge research would benefit from the wider adoption and continued development of adolescent-specific and sport-specific validated instruments to improve confidence in the interpretation and comparability of reported knowledge scores.

Future intervention research should place greater emphasis on achieving sustained dietary behaviour change rather than improving nutrition knowledge alone. The current evidence suggests that education-only approaches are often insufficient, particularly when interventions do not account for the broader social and environmental contexts that shape food choice in youth athletes. Future studies should evaluate multicomponent approaches that integrate behaviour change strategies with practical support that addresses individual, social, environmental and cultural influences on dietary behaviour. More consistent reporting of intervention content, behaviour change techniques, outcome measures and follow-up duration is needed to help identify which components, or combinations of components, are most effective in producing sustained behaviour change across different populations and settings. At present, most studies did not assess outcomes beyond the immediate post-intervention period, making it difficult to determine whether observed effects represent lasting change. Further research is also needed to examine how the factors influencing dietary behaviour in youth athletes may evolve across adolescence and differ by gender, so that interventions can be designed to reflect the real-world contexts in which dietary choices are made.

Given the scope and persistence of the methodological inconsistencies identified in this review, the development of an expert consensus statement on minimum reporting and methodological standards for nutrition research in youth team sport athletes may be warranted. Such a statement could consolidate current best practices across dietary assessment, nutrition knowledge measurement, intervention design and participant characterisation into a single reference framework. Rather than prescribing a single methodological approach, it could provide decision-based guidance to help researchers navigate the range of available methods and select those most appropriate for their study design, sample size and available resources, while establishing minimum standards for transparency and rigour. A consolidated resource of this kind does not currently exist for this population, and its development could meaningfully improve the quality, comparability, and practical value of future research in this field. In the interim, this review provides two summary tables to support both future research and applied practice. Table 7 outlines key methodological limitations and recommendations to guide future research design, while Table 8 translates the review's findings into applied considerations for practitioners working with youth team sport athletes.

### Limitations

This scoping review has several limitations that should be considered when interpreting its findings. The exclusion of studies focusing solely on vitamin or supplement use may have omitted relevant insights into athletes' micronutrient status. The inclusion of only English-language publications introduces potential language bias, which may have narrowed the evidence base. Considerable heterogeneity in study designs, dietary assessment methods and nutrition knowledge tools limited comparability across studies, although such variability is expected in scoping reviews and was a central finding of this review rather than an incidental limitation. As a scoping review, this study was designed to map and synthesise the available evidence rather than evaluate efficacy or establish causality and its findings should be interpreted accordingly. Despite these limitations, this review provides a comprehensive synthesis of the available evidence, identifies persistent methodological gaps and offers recommendations to advance both research and practice in this field.

## Conclusion

This scoping review synthesised evidence on dietary intake, nutrition knowledge, education interventions and factors influencing dietary behaviours in youth team sport athletes. While informative, the strength of the evidence was constrained by methodological inconsistencies, limited female representation and widespread reliance on self-reported dietary data, much of which lacked adjustment for misreporting. Inadequate energy intake was commonly reported, with deficits most apparent on training and match days. However, findings from studies using doubly labelled water to assess energy expenditure suggest that the magnitude of these deficits may be overestimated due to dietary underreporting and imprecise estimation of energy expenditure. Reported carbohydrate intake frequently fell below recommended levels, reflecting inadequate alignment with training demands and contributing to suboptimal energy availability. Protein and fat intake generally met or exceeded recommendations, although concerns regarding excessive saturated fat intake and limited assessment of fat quality were noted. Micronutrient intake often fell short of recommendations, particularly for vitamins A, D and E, calcium, and iron, likely reflecting low fruit and vegetable consumption. Nutrition knowledge was limited, particularly for sport-specific topics, and none of the questionnaires used to assess knowledge were validated for adolescent populations. Education interventions varied widely in design and effectiveness, with short-term improvements in knowledge rarely translating into sustained behavioural change. Interventions that incorporated behaviour change components beyond education delivery more often reported improvements in dietary behaviour, although the limited number of studies precludes firm conclusions. Dietary behaviours were shaped by a complex interplay of individual, social and environmental influences, reinforcing the need for approaches that extend beyond education alone.

Key recommendations for future research and practice are presented in the preceding section and in Tables 7 and 8. Given the scope and persistence of the methodological inconsistencies identified in this review, the development of an expert-led consensus statement on standards for dietary assessment, nutrition knowledge measurement, intervention design and participant characterisation may help advance the quality and comparability of future research in this population.

**Table 7.** Methods limitations and research recommendations identified in this scoping review.

Thematic category	Identified limitation	Recommendation for future research	Rationale
Study Design	Predominant use of cross-sectional designs	Prioritise longitudinal, repeated-measures, and intervention designs	Enables tracking developmental changes in dietary intake, nutrition knowledge and behaviour over time
Study Design	Limited use of statistical power analyses	Report a priori power analyses and sample size justifications in study protocols	Supports transparency, improves precision of effect estimates, and reduces Type II error risk; underpowered studies also inflate the proportion of false positives among significant findings, increasing Type I error rate
Study Design	Lack of follow-up in intervention studies	Include follow-up assessments to evaluate knowledge retention and behaviour change	Determines if effects persist post-intervention
Study Design	Use of adult-based frameworks to classify athlete calibre	Develop and adopt youth-specific athlete classification systems	Ensures consistent athlete classification and relevance for youth sport populations
Participant Representation	Under-representation of female athletes and limited sex-specific analysis	Aim for gender-balanced samples and consider sex-stratified analyses where appropriate	Enhances inclusivity and supports interpretation of sex-specific differences in nutritional needs and behaviour
Dietary Assessment	Inconsistent reporting of dietary intake data	Where feasible, report dietary intake using standard units (e.g., mean kcal/day, g/kg/day)	Facilitates cross-study comparability
Dietary Assessment	Limited adjustment for misreporting in self-reported dietary data	Report whether implausible dietary intake values were assessed, apply bias correction methods where possible (e.g., Goldberg cut-offs), and describe the validation strategies used	Improves the accuracy of dietary intake estimates, particularly in adolescent populations vulnerable to recall and reporting bias
Dietary Assessment	Reliance on self-reported dietary intake and estimated energy expenditure to assess energy availability without acknowledging measurement limitations	Acknowledge the limitations of field-based energy availability estimates and, where possible, report both unadjusted and adjusted values to account for potential misreporting	Evidence from doubly labelled water studies suggests that the magnitude of energy deficits may be overestimated; transparent reporting improves interpretive confidence
Dietary Recommendations	Use of adult dietary recommendations in youth sport populations	Develop and validate age-appropriate dietary guidelines for youth athletes	Youth athletes have unique growth and metabolic demands, making adult recommendations potentially inaccurate
Nutrition Knowledge	Lack of standardisation in nutrition knowledge assessment tools	Use validated and, if feasible, adolescent-specific instruments	Improves population relevance and reliability, enabling consistent mapping of nutrition knowledge across studies
Intervention design	Limited use and inconsistent reporting of behaviour change components in education interventions	Report behaviour change techniques used, alongside intervention content, delivery format, and follow-up duration	Enables identification of which components, or combinations of components, are most effective in producing sustained dietary behaviour change
Behavioural influences	Intervention studies rarely isolated the effect of specific behaviour change techniques, typically combining multiple components without adequate control conditions to attribute outcomes to individual components	Where feasible, use dismantling designs or active control conditions, such as education-only versus education combined with a specific behaviour change technique, to identify which components drive dietary behaviour change	While dietary behaviour is shaped by multiple interwoven influences, isolating the contribution of individual behaviour change techniques where feasible would improve understanding of which components drive change, enabling more targeted and evidence-informed intervention design

**Table 8.** Key applied insights for practitioners working with youth team sport athletes.

Identified insight	Practical recommendation	Implication(s) for practice
The predominance of observational designs limits understanding of how dietary needs and behaviours evolve during adolescence	Practitioners should reassess athletes’ nutritional needs at different growth stages and training cycles	Given the rapid developmental changes in adolescence, adaptive nutrition strategies are essential to support optimal health, recovery and performance
Energy deficits are often reported on match days and during periods of high training loads, but these may be overestimated due to under-reporting	Practitioners should assess energy intake during demanding periods and align fuelling strategies with individual exercise demands	Unaddressed energy deficits may compromise growth, recovery and performance and increase injury risk
Carbohydrate intake in youth athletes is often insufficient and poorly aligned with training demands	Consider supporting athletes in aligning carbohydrate intake with the demands of training and competition through individualised, practical guidance	Optimising carbohydrate intake may support glycogen replenishment, recovery and energy availability
Protein intake generally met or exceeded recommendations but frequently coexisted with inadequate overall energy intake	Practitioners should evaluate protein intake within the context of overall energy and macronutrient adequacy rather than in isolation	Adequate protein intake may mask broader dietary inadequacy when energy and carbohydrate intake are suboptimal
Fat intake generally met proportional guidelines, but absolute intake may be inadequate during periods of low energy availability and excessive saturated fat intake was a common concern	Consider assessing both the quantity and quality of dietary fat intake, including the balance of saturated, monounsaturated and polyunsaturated sources	A focus on fat quality alongside quantity may better support long-term health and dietary adequacy in youth athletes
This review identified frequent dietary shortfalls in key micronutrients, including vitamins A, D and E, calcium, and iron, alongside low fruit and vegetable consumption	Encourage a varied diet rich in fruits, vegetables, dairy and be aware of the micronutrient shortfalls most commonly reported in this population when providing dietary guidance	Promoting dietary patterns that support adequate micronutrient intake through food-first strategies may support bone health, immune function and overall development during adolescence
Nutrition knowledge was generally limited among youth team sport athletes, particularly in areas such as supplementation, nutrient timing and micronutrient function	Deliver age-appropriate nutrition education that addresses the specific knowledge gaps identified in this population, with consideration for practical application rather than theoretical knowledge alone	While improved knowledge alone may not be sufficient to change dietary behaviour, it provides a necessary foundation for informed decision-making
Dietary behaviours in youth athletes are shaped by individual, social and environmental factors, and education alone rarely produced sustained dietary change	Assess the individual, social, environmental and cultural factors shaping each athlete's dietary practices to inform targeted and realistic nutrition support	Nutrition advice is more likely to be effective and sustained when it accounts for the real-world contexts in which youth athletes make dietary choices and is reinforced by key people in their environment

The recommendations presented in this table are informed by a body of literature with notable methodological constraints. They are intended to provide directional guidance rather than prescriptive practice standards, and should be interpreted in light of the limitations outlined in Table 7 and the Limitations section of this review.

## Appendix A. Database search strings.

PubMed and SPORTDiscus Search Strategy.

("dietary intervention*" OR "dietary education" OR "eating behaviour*" OR "eating behaviour*" OR "education intervention*" OR "educational intervention*" OR "nutrition intervention*" OR "nutritional intervention*" OR "nutrition programme*" OR "nutritional programme*" OR "nutrition education" OR "nutritional education" OR "food education" OR "food intervention" OR "nutrition knowledge" OR "nutritional knowledge" OR "nutrition* literacy" OR "food knowledge" OR "general nutrition knowledge" OR "nutrition for sport knowledge questionnaire" OR "Nutrition knowledge questionnaire" OR "nutrition attitude*" OR "food attitude*" OR "nutrition belief*" OR "food belief*" OR "nutritional preference*" OR "food environment" OR "nutritional priorities" OR "nutrition priorities" OR "nutrition barriers" OR "nutritional barriers" OR "cognitive behavioural therapy" OR "cognitive behavioural therapy" OR "CBT" OR "behaviour change*" OR "behaviour change*" OR "food habits" OR "nutrition* habits" OR "habit formation" OR "behaviour change model" OR "behaviour change model" OR "behaviour change techniques" OR "behaviour change techniques" OR "nutrition behaviour*" OR "nutrition behaviour*" OR "nutritional behaviour*" OR "nutritional behaviour*" OR "nutrient status" OR "nutritional status" OR "nutrient intake" OR "nutrient requirement" OR "dietary intake*" OR "daily food" OR "food-diary" OR "food intake*" OR "nutrient intake*" OR "nutrients intake" OR "dietary assessment" OR "diet analysis" OR "dietary analysis" OR "dietary habits" OR "sports nutrition" OR "sport nutrition" OR "nutrition" OR "RDA" OR "calcium intake" OR "carbohydrate" OR "carbohydrates" OR "carbohydrate intake*" OR "energy availability" OR "energy balance" OR "energy intake" OR "macronutrient" OR "macronutrients" OR "micronutrient" OR "micronutrients" OR "macronutrient intake" OR "micronutrient intake" OR "protein" OR "protein intake" OR "fat intake" OR "iron status" OR "iron intake" OR "calorie intake" OR "food consumption" OR "recommended daily allowance" OR "cooking skills" OR "cooking" OR "cooking literacy" OR "food literacy" OR "cooking practices" OR "food skills" OR "meal preparation" OR "food preparation").

AND

("field sport" OR "field-sports" OR "field-based sport" OR "field based sport" OR "field based sports" OR "field-sport athlete" OR "field sport athlete" OR "field sports athletes" OR "team-sport" OR "team sport" OR "team sports" OR "team based sport" OR "team sport players" OR "team sport athletes" OR "collision team sports" OR "collision sport" OR "collision sports" OR "collision sport athlete" OR "collision sport athletes" OR "Rugby" OR "Rugby league" OR "Basketball" OR "Soccer" OR "Football" OR "Australian Rules" OR "Australian Football" OR "Hurling" OR "Camogie" OR "Hockey" OR "Field Hockey" OR "Lacrosse" OR "Gaelic" OR "Gaelic games" OR "Gaelic Football").

PsycINFO and Medline Search Strategy.

("dietary intervention*" OR "nutrition intervention*" OR "nutritional intervention*" OR "nutrition knowledge" OR "nutritional knowledge" OR "behaviour change*" OR "behaviour change*" OR "nutrient intake" OR "dietary intake*").

AND

("team-sport" OR "team sport" OR "team sports" OR "team based sport").
